# Endothelin-1 as a Candidate Biomarker of Systemic Sclerosis: A GRADE-Assessed Systematic Review and Meta-Analysis With Meta-Regression

**DOI:** 10.1177/11772719251318555

**Published:** 2025-02-21

**Authors:** Arduino A Mangoni, Angelo Zinellu

**Affiliations:** 1 Discipline of Clinical Pharmacology, College of Medicine and Public Health, Flinders University, Adelaide, SA, Australia; 2 Department of Clinical Pharmacology, Flinders Medical Centre, Southern Adelaide Local Health Network, Adelaide, SA, Australia; 3 Department of Biomedical Sciences, University of Sassari, Sassari, Italy

**Keywords:** Endothelin-1, systemic sclerosis, biomarkers, fibrosis, vascular dysfunction, organ involvement

## Abstract

**Background:**

There is an ongoing search for novel biomarkers of vascular dysfunction, extent of fibrosis and organ involvement in systemic sclerosis (SSc).

**Objectives:**

We critically appraised the studies investigating the circulating concentrations of endothelin-1 in SSc patients and healthy controls.

**Design:**

This was a systematic review with meta-analysis.

**Data sources and methods:**

We searched electronic databases (PubMed, Scopus, and Web of Science) from inception to 15 June 2024. We assessed the risk of bias and the certainty of evidence using the JBI Critical Appraisal Checklist and GRADE, respectively.

**Results:**

Endothelin-1 concentrations were significantly higher in SSc patients than in controls (26 studies; standardised mean difference, SMD = 0.98, 95% CI 0.73-1.23, *P* < .001; moderate certainty of evidence). In SSc patients, there were no significant differences in endothelin-1 concentrations between those with limited and diffuse cutaneous SSc (10 studies; SMD = 0.32, 95% CI −0.07 to 0.71 *P* = .11; very low certainty), and with and without digital ulcers (5 studies; SMD = 0.82, 95% CI −0.06 to 1.69, *P* = .066; very low certainty), pulmonary arterial hypertension (7 studies; SMD = 0.22, 95% CI −0.01 to 0.45, *P* = .066; very low certainty) or interstitial lung disease (3 studies; SMD = 0.09, 95% CI −0.18 to 0.35, *P* = .51; very low certainty). There was limited evidence in SSc patients with different video capillaroscopy pattern and telangiectasias. Subgroup and meta-regression analyses showed significant associations between the effect size and geographical location (studies investigating SSc patients and controls), year of publication (studies investigating SSc patients with limited and diffuse cutaneous SSc), and biological matrix assessed (studies investigating SSc patients with and without digital ulcers).

**Conclusion:**

The results of this systematic review and meta-analysis highlight the potential role of endothelin-1 as a candidate biomarker of SSc. Further research is warranted to determine the utility of measuring endothelin-1 in SSc subgroups with different extent of fibrosis and organ involvement.

**Registration:**

PROSPERO registration number - CRD42024566461.

## Introduction

Vascular dysfunction is a common feature of systemic sclerosis (SSc), an autoimmune condition characterised by excess fibrosis affecting the skin and other organs.^1-4^ Vascular and microvascular abnormalities account for several disabling clinical manifestations in SSc, including the Raynaud’s phenomenon, telangiectasias, digital ulcers and pulmonary arterial hypertension.^5-9^ Furthermore, there is a complex yet recognised interplay between vascular dysfunction and excess fibrosis in SSc.^10-12^ This suggests that the availability of biomarkers reflecting these processes may be useful in the clinical assessment and monitoring of this patient group.

The family of endothelins, which includes 3 similar endogenous 21-amino acid peptides (endothelin-1, endothelin-2 and endothelin-3), has been extensively studied in various disease states in virtue of their vasoconstrictive, pro-angiogenic, and pro-fibrotic effects.^13^ Endothelin-1, the first identified and most abundant endothelin, is primarily synthesised in the endothelium and exerts its effects by binding to the G-protein coupled receptors, ET_A_ and ET_B_.^14^ The main regulators of the release of endothelin-1 from endothelial cells include shear stress, hypoxia, thrombin, angiotensin II and transforming growth factor β (TGF-β).^13,15^ The vasoconstrictive effects of endothelin-1 are primarily mediated by the ET_A_ receptor, which also promotes the proliferation of vascular smooth muscle cells.^16^ By contrast, ET_B_ activation mediates vasodilation through maintaining a basal release of the endogenous messenger, nitric oxide.^14,17^ Endothelin-1 has also been shown to promote angiogenesis in cancer as a critical mechanism favouring tumour growth and metastasis. By promoting hypoxia in the tumour microenvironment,^18^ endothelin-1 can activate the heterodimer hypoxia-inducible factor-1 (HIF-1), with consequent upregulation of the vascular endothelial growth factor (VEGF).^19-21^ Furthermore, there is good evidence that endothelin-1 can promote fibrosis in several organs, including the skin, lung, heart, and liver.^22-26^ The pro-fibrotic effects of endothelin-1 have also been investigated in SSc. Experimental studies have shown that the dysregulation of the TGF-β/endothelin-1/Ras signalling pathway, with overexpression of the associated genes, plays a critical role in the pathophysiology of SSc through the activation of fibroblast function and progression of fibrosis.^27,28^ Furthermore, studies have elegantly reported that endothelin-1 induces the polarisation of macrophages towards a pro-fibrotic M2 phenotype. This process can significantly contribute per se to the pathogenesis and the progression of SSc.^29^

Studies have generally reported elevations in circulating endothelin-1 in SSc patients, particularly in those with digital ulcers, specific video capillaroscopy patterns (ie, active pattern) and organ involvement (ie, renal crisis and pulmonary arterial hypertension).^30^ However, other studies have reported lower circulating endothelin-1 in other SSc subgroups (ie, patients with early video capillaroscopy pattern).^30^ The results of these studies, together with the established therapeutic role of endothelin receptor antagonists in patients with specific organ involvement,^31^ suggest that this peptide may be a candidate biomarker of SSc.

We investigated this issue by conducting a systematic review and meta-analysis of studies investigating endothelin-1 concentrations in SSc patients and healthy controls as well as SSc patients with different extent of fibrosis, nailfold video capillaroscopy pattern and organ involvement. We also assessed possible associations between the effect size of the between-group differences in endothelin-1 concentrations and several study and patient characteristics.

## Methods

### Search strategy and study selection

We conducted a literature search in the electronic databases, PubMed, Web of Science and Scopus from inception to 15 June 2024. We used the following terms to identify relevant articles (please refer to Supplemental Table 1 for the details of the search strategy in each database): ‘systemic sclerosis’ OR ‘scleroderma’ OR ‘SSc’ AND ‘endothelin’ OR ‘endothelin-1’ OR ‘ET-1’. Two investigators independently screened each abstract. If the abstract was considered relevant, the investigators independently reviewed the full text of the article.

The inclusion criteria were: (i) the measurement of endothelin-1 concentrations in patients with SSc diagnosed according to accepted criteria and healthy controls, and in SSc patients with limited or diffuse cutaneous SSc, specific nailfold video capillaroscopy pattern (early, active and late),^32^ and organ complications, (ii) case-control studies, (iii) the recruitment of adult participants and (iv) the availability of the full text of the publication in English language. The exclusion criteria were: (i) animal studies, (ii) recruitment of participants under 18 years and (iii) non-case-control studies. The reference list of each article was hand searched for additional studies.

Two investigators independently extracted the following data from each article: year of publication, first author, country and continent where the study was conducted, sample size, age, male-to-female ratio, endothelin-1 concentrations, mean disease duration, biological matrix assessed (serum or plasma), analytical method used and proportion of patients with limited or diffuse cutaneous SSc and specific complications (eg, digital ulcers, pulmonary arterial hypertension and telangiectasias).

We assessed the risk of bias for each article using the Joanna Briggs Institute (JBI) Critical Appraisal Checklist for analytical studies,^33^ and the certainty of evidence using the Grades of Recommendation, Assessment, Development, and Evaluation (GRADE) Working Group system.^34^ The risk of bias for each article was evaluated to detect the presence of methodological flaws that could affect the interpretation of the results of the meta-analysis as well as the level of the certainty of evidence. Specifically, the domains assessed for each article included: clear definition of inclusion criteria, clear description of participant characteristics and study setting, reliability in the measurement of the exposure, use of standard criteria to assess the condition, consideration of confounding factors and use of relevant statistical methods, reliable outcome measurement, and overall appropriate use of statistical methods.^33^ Studies addressing ⩾75%, ⩾50% and <75% and <50% of these criteria were considered as having low, moderate, and high risk of bias, respectively. We followed the Preferred Reporting Items for Systematic Reviews and Meta-Analyses 2020 statement,^35^ and registered the study in the International Prospective Register of Systematic Reviews (PROSPERO registration number: CRD42024566461).

### Statistical analysis

We generated forest plots of standardised mean differences (SMDs) and 95% confidence intervals (CIs) to assess between-group differences in endothelin-1 concentrations (a *P*-value < .05 was considered statistically significant). When necessary, we extracted data from graphs using the Graph Data Extractor software (San Diego, CA, USA), and extrapolated means and standard deviations from medians and interquartile or full ranges using published methods.^36^ SMD heterogeneity was assessed using the Q statistic (the significance level was set at a *P*-value < .10), and ranked as low (*I*^2^ ⩽25%), moderate (25%< *I*^2^ <75%), or high (*I*^2^ ⩾75%). We used a random-effects model based on the inverse-variance method in presence of high heterogeneity.^37,38^ We conducted sensitivity analyses to confirm the stability of the results.^39^ We assessed potential publication bias, related to small study effects and/or the tendency for smaller studies with significant or positive results to be published more frequently than those with null or negative outcomes, using Begg’s adjusted rank correlation test, Egger’s regression asymmetry test (a *P*-value < .05 was considered statistically significant) and the ‘trim-and-fill’ method.^40-42^ We conducted univariate meta-regression and subgroup analyses to investigate associations between the effect size and study and patient characteristics and to identify sources of heterogeneity. We used Stata 14 for all statistical analyses (Stata Corp., College Station, TX, USA).

## Results

**Figure 1.**
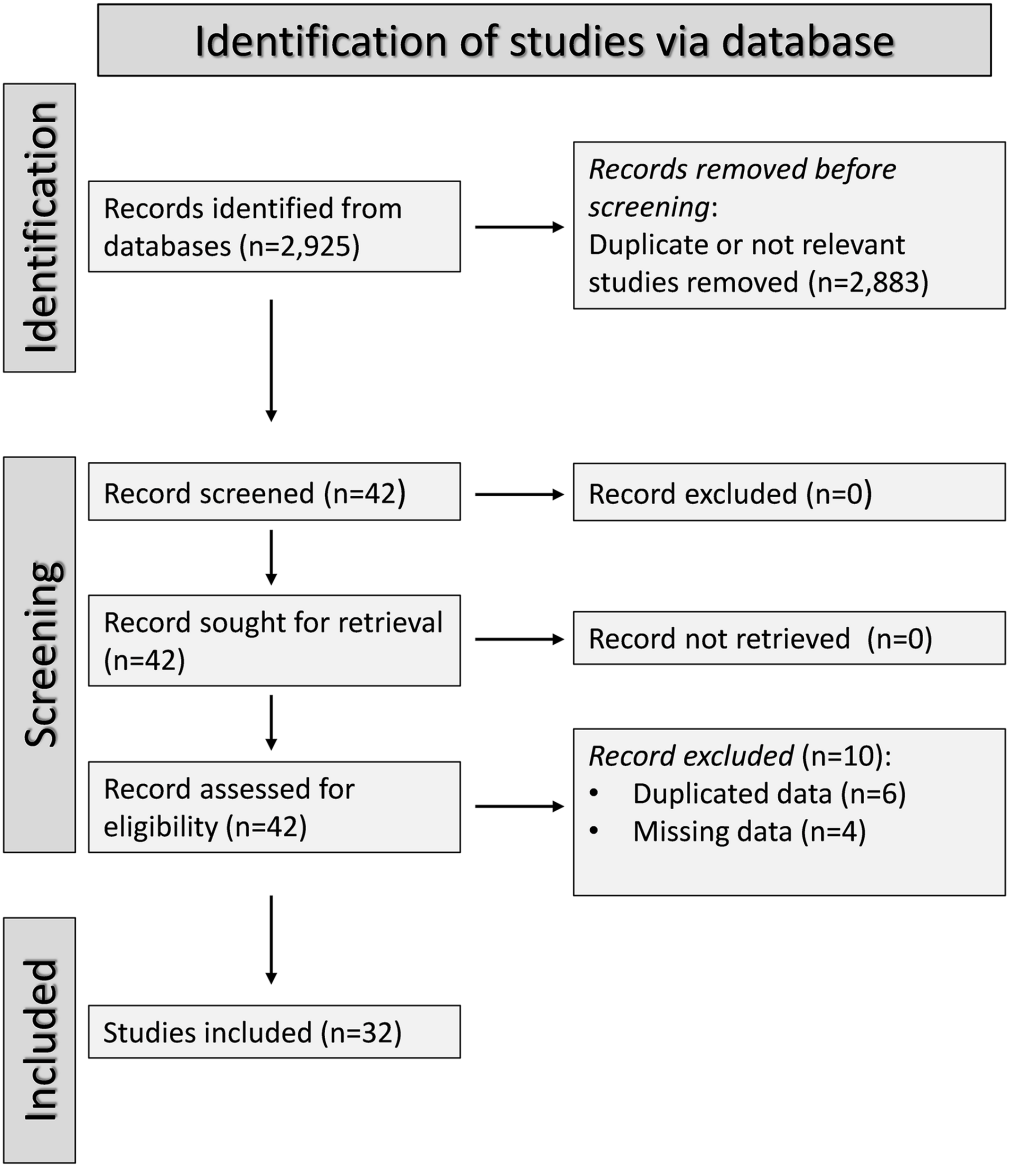
Flow diagram of study selection.

The flow chart (Figure 1) shows that, from a total of 2925 articles identified, 2883 were excluded after the initial screening because they presented duplicate or irrelevant data. A full-text review of the remaining 42 articles led to the further exclusion of 10 studies because of duplicate data (n = 6) or using a non-case-control design (n = 4). Therefore, 32 studies (Table 1) were included in the analysis.^43-74^ Assessment of the risk of bias (Supplemental Table 2) showed that 19 studies had a low risk,^43,46,48,51,53,55,57,59,61,63-67,69-73^ and the remaining 13 a moderate risk.^44,45,47,49,50,52,54,56,58,60,62,68,74^ The cross-sectional design of the selected studies accounted for the low initial level of the certainty of evidence (level 2).

**Table 1.** Characteristics of studies investigating endothelin concentrations in patients with systemic sclerosis and healthy controls.

Study	Matrix	Controls				Patients with systemic sclerosis				
		n	Age (years)	M/F	Endothelin (mean ± SD)	n	Age (years)	M/F	Endothelin (mean ± SD)	MDD (years)
Yamane K et al, 1992 Japan^43^	P	25	matched	matched	1.31 ± 0.1	31	56.8	3/28	1.9 ± 0.47	5.25
Kadono T et al, 1995, Japan^45^	P	21	50	matched	7.4 ± 8.6	64	55	matched	28.1 ± 36.9	15.3
Morelli S et al, 1995, Italy^46^	P	18	45	3/15	0.63 ± 0.19	48	47.2	7/41	1.65 ± 0.29	NR
Maeda M et al, 1997, Japan^47^	P	10	45.6	0/10	1.31 ± 0.34	43	48.1	7/36	1.99 ± 0.57	NR
Silveri F et al, 2001, Italy^48^	P	13	NR	NR	3.22 ± 2.31	17	NR	NR	11.6 ± 8.5	NR
Fontana F et al, 2005, Italy^49^	P	20	NR	10/10	0.4 ± 0.18	14	NR	5/9	0.41 ± 0.19	NR
Kuryliszyn-Moskal A et al, 2005, Poland^50^	S	30	matched	matched	4.28 ± 2.63	31	55.2	0/31	17.33 ± 8.75	7.8
Paterlana D et al, 2009, Italy^52^	S	75	57.8	5/70	3.98 ± 3.13	82	55.5	5/77	5.48 ± 2.95	NR
Coral-Alvarado P et al, 2009, Colombia^51^	S	20	NR	matched	0.166 ± 0.067	20	54.4	6/14	0.202 ± 0.057	9.35
Sulli A et al, 2009, Italy^53^	P	45	53	9/36	1.3 ± 1.3	99	60	9/90	2.1 ± 1.6	6
Kim HS et al, 2010, Korea^54^	P	30	35.7	0/30	1.57 ± 1.56	60	41.7	8/52	3.2 ± 4.1	7.3
Pehlivan Y et al, 2011, Turkey^55^	P	30	matched	matched	1.23 ± 1.59	55	51.3	4/51	6.39 ± 10.01	6.36
Michaelis T et al, 2012, Brazil^57^	S	37	49.57	3/34	2.65 ± 1.23	37	48.97	3/34	2.51 ± 1.98	42.5
Irzyk K et al, 2013, Poland^59^	S	21	49.3	3/18	1.3 ± 0.6	111	54.2	10/101	1.9 ± 1.4	9.12
Penn et al, (a) 2013, UK^60^	S	20	56	NR	0.503 ± 0.513	27	52.8	NR	0.878 ± 1.438	11.6
Penn et al, (b) 2013, UK^60^	S	20	56	NR	0.503 ± 0.513	26	61.2	NR	3.03 ± 5.204	20
Kawashiri S et al, 2014, Japan^62^	P	15	42	7/8	0.533 ± 0.659	24	60	4/20	2.9 ± 2.13	13
Yilmaz N et al, 2014, Turkey^63^	S	16	39.8	matched	0.58 ± 0.29	55	44.5	3/52	3.43 ± 6.05	6.6
Camargo CZ et al, 2015, Brazil^61^	S	45	48.4	6/39	1.27 ± 0.73	126	50.4	14/112	1.82 ± 0.73	6.9
Silva I et al, 2015 Portugal^64^	P	40	matched	matched	2.69 ± 4.31	77	52.95	5/72	12.56 ± 6.53	NR
Benyamine et al, 2017, France^65^	S	41	56.09	3/38	1.17 ± 0.23	45	61.49	1/44	1.66 ± 0.54	NR
Hajialilo M et al, 2017, Iran^66^	S	60	47.2	9/51	0.33 ± 0.09	60	46.2	6/54	0.6 ± 0.27	6.62
Nicola S et al, 2017, Italy^67^	S	20	59	8/12	5.2 ± 0.9	29	64.2	1/28	20.9 ± 11.2	NR
Al-Omary Obadeh M et al, 2021, Ukraine^69^	P	35	39.5	13/22	3.59 ± 1.42	78	43.2	27/51	8.47 ± 2.77	NR
Stochmal A et al, 2021, Poland^70^	S	20	matched	matched	1.31 ± 0.49	100	58.3	6/94	1.99 ± 1.38	8.74
Apti Sengun O et al, 2023, Turkey^71^	S	23	41.3	5/18	50.8 ± 24.5	24	42.8	4/20	103 ± 104	NR
Bhattacharjee D et al, 2023, India^72^	S	20	35.3	4/16	8.01 ± 3.37	56	35.4	10/46	10.6 ± 3.77	3.5

Abbreviations: MDD, mean disease duration; M/F, male to female ratio; NR, not reported; P, plasma; S, serum.

### Endothelin-1 and presence of systemic sclerosis

As reported in Table 1, 26 studies, including 27 group comparators, investigated endothelin-1 in 1439 SSc patients (mean age 52 years, 89% females) and 770 healthy controls (mean age 50 years, 83% females).^43,45-55,57,59-67,69-72^ Thirteen studies were conducted in Europe,^46,48-50,52,53,59,60,64,65,67,69,70^ ten in Asia,^43,45,47 54,55,62,63,66,71,72^ and three in America.^51,57,61^ Endothelin-1 was measured in plasma in 12 studies,^43,45-49,53-55,62,64,69^ and in serum in 14 studies.^50-52,57,59-61,63,65-67,70-72^ An enzyme-linked immunosorbent assay was used in 21 studies,^43,45,47,50-55,57,59-63,65,66,69-72^ a radioimmunoassay in 4 studies,^46,48,49,64^ and a platform for multi-analyte profiling in one study.^67^ Mean disease duration, reported in 16 studies, ranged between 3.5 and 42.5 years.^43,45,50,51,53-55,57,59-63,66,70,72^ SSc patients received treatment with endothelin receptor antagonists in 3 studies.^60,66,71^ The risk of bias (Supplemental Table 2) was low in 18 studies,^43,46,48,51,53,55,57,59,61,63-67,69-72^ and moderate in the remaining eight.^45,47,49,50,52,54,60,62^

**Figure 2.**
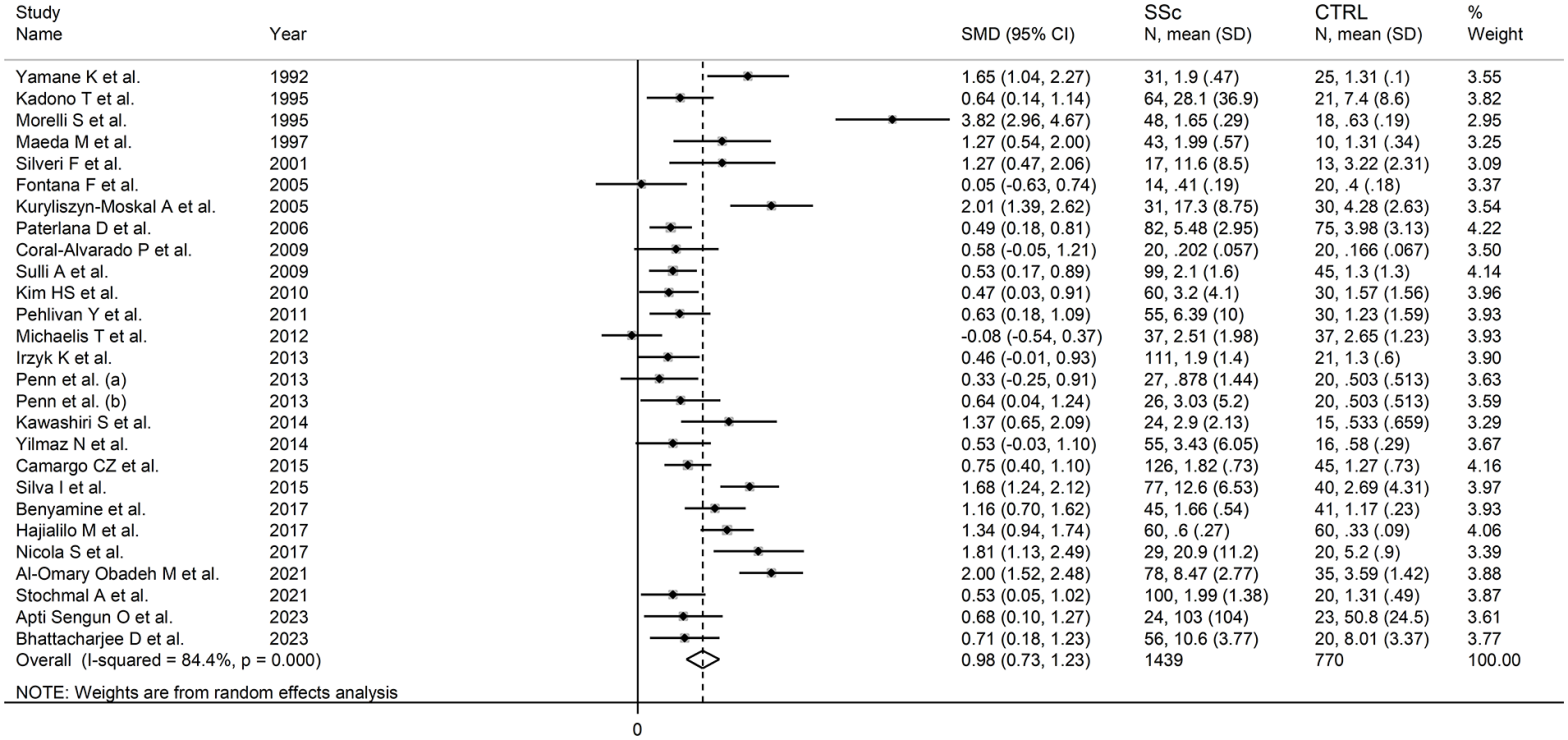
Forest plot of studies measuring endothelin in patients with systemic sclerosis and healthy controls.

**Figure 3.**
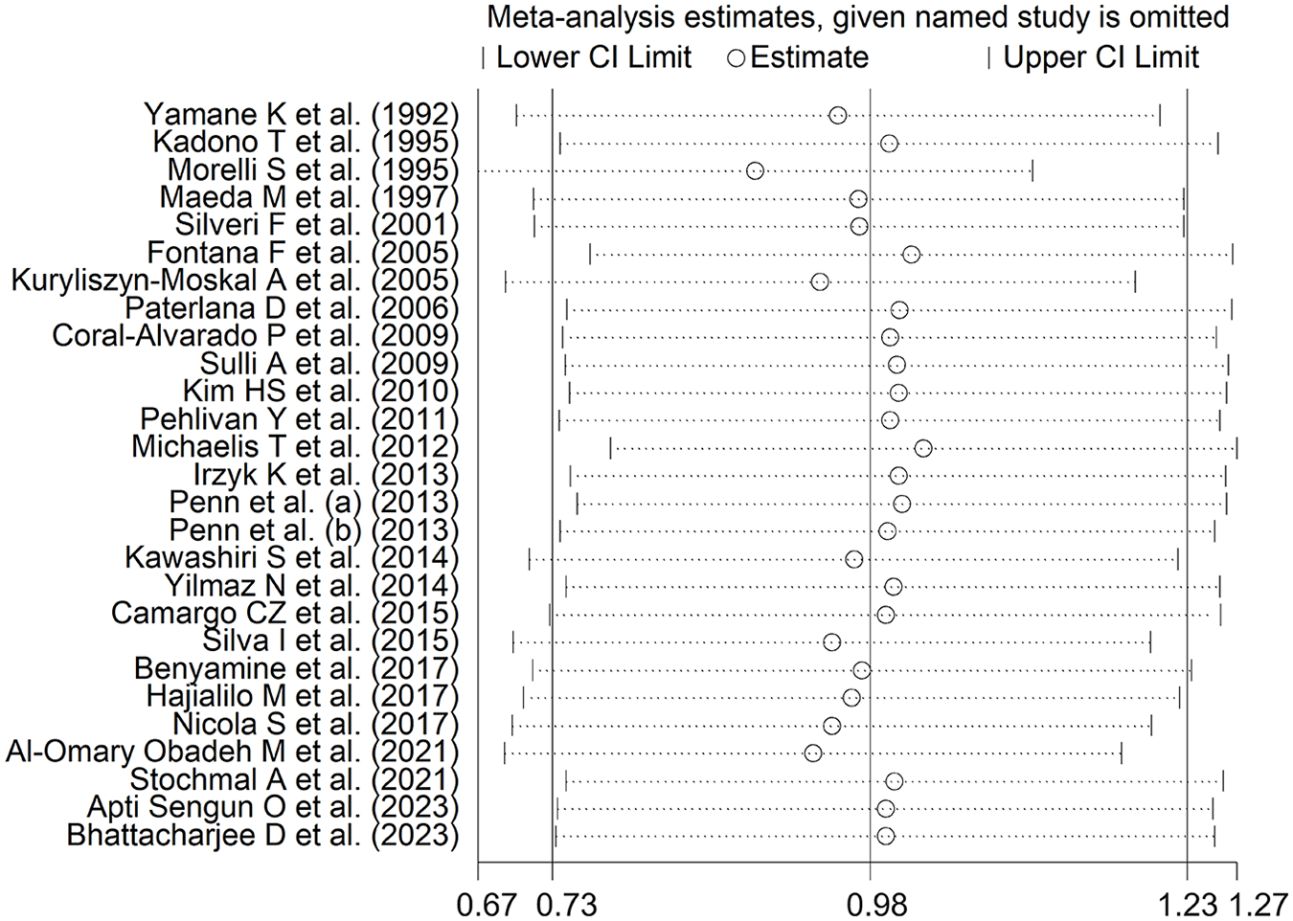
Sensitivity analysis of the association between endothelin and systemic sclerosis.

The forest plot (Figure 2) showed that SSc patients had significantly higher endothelin-1 concentrations than controls (SMD = 0.98, 95% CI 0.73-1.23, *P* < .001; *I*^2^ = 84.4%, *P* < .001). The pooled SMD values were stable in sensitivity analysis, ranging between 0.89 and 1.02 (Figure 3).

**Figure 4.**
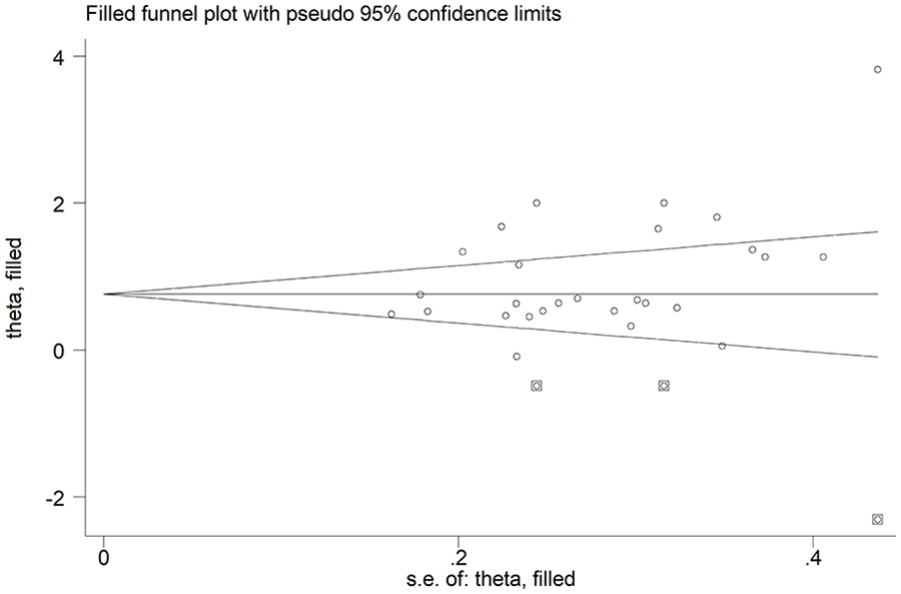
Funnel plot of studies investigating endothelin in systemic sclerosis using the ‘trimming-and-filling’ method. The enclosed circles and free circles represent the dummy and genuine studies, respectively.

We did not observe a significant publication bias according to Begg’s (*P* = .045) and Egger’s test (*P* = .054). The ‘trim-and-fill’ method identified 3 missing studies to be added to the left side of funnel plot to ensure symmetry (Figure 4). The resulting effect size was mildly attenuated yet still significant (SMD = 0.80, 95% CI 0.51-1.08, *P* < .001).

We did not observe any significant associations in meta-regression analysis between the effect size and age (*t* = 0.31, *P* = .76), male-to-female ratio (*t* = −0.74, *P* = .47), year of publication (*t* = −1.27, *P* = .22), number of participants (*t* = −0.56, *P* = .58), mean disease duration (*t* = −1.98, *P* = .066), or the proportion of patients with diffuse disease (*t* = 1.07, *P* = .30), interstitial lung disease (*t* = −0.17, *P* = .87), and pulmonary arterial hypertension (*t* = −0.15, *P* = .88). In sub-group analysis, we observed that the pooled SMD was significantly different in European (SMD = 1.16, 95% CI 0.74-1.59, *P* < .001; *I*^2^ = 89.5%, *P* < .001) and Asian studies (SMD = 0.90, 95% CI 0.64-1.17, *P* < .001; *I*^2^ = 58.8%, *P* = .009), but not in American studies (SMD = 0.42, 95% CI −0.13 to 0.97, *P* = .14; *I*^2^ = 76.0%, *P* = .016; Supplemental Figure 1), with a lower between-study variance in the Asian subgroup. There was no significant difference (*P* = .13) in the pooled SMD between studies assessing serum (SMD = 0.78, 95% CI 0.52-1.04, *P* < .001; *I*^2^ = 75.7%, *P* < .001) and plasma (SMD = 1.25, 95% CI 0.78-1.72, *P* < .001; *I*^2^ = 88.7%, *P* < .001; Supplemental Figure 2). There was a trend towards a significantly higher (*P* = .058) pooled SMD in studies using a radioimmune assay (SMD = 1.69, 95% CI 0.38-3.00, *P* = .007; *I*^2^ = 93.5%, *P* < .001) compared to studies using an enzyme-linked immunosorbent assay (SMD = 0.83, 95% CI 0.61-1.05, *P*< .001; *I*^2^ = 76.9%, *P* < .001; Supplemental Figure 3), with a lower between-study variance in the latter subgroup. There was no significant difference (*P* = .47) in the pooled SMD between studies using an enzyme-linked immunosorbent assay on plasma (SMD = 1.04, 95% CI 0.62-1.47, *P* < .001; *I*^2^ = 82.0%, *P* < .001), radioimmune assay on plasma (SMD = 1.69, 95% CI 0.38-3.00, *P* = .007; *I*^2^ = 93.5%, *P* < .001), and enzyme-linked immunosorbent assay on serum (SMD = 0.72, 95% CI 0.47-0.96, *P* < .001; *I*^2^ = 72.8%, *P* < .001; Supplemental Figure 4), with a lower between-study variance in the latter subgroup.

We upgraded the final level of the certainty of evidence to moderate (level 3) after considering the low-moderate risk of bias in all studies (no change), the high but partially explainable heterogeneity (no change), the lack of indirectness (no change), the large effect size (SMD = 0.98, upgrade 1 level),^75^ and the presence of publication bias which was addressed using the ‘trim-and-fill’ method (no change).

### Endothelin-1 and extent of fibrosis

Ten studies (Table 2) investigated endothelin-1 in 260 SSc patients with diffuse cutaneous SSc and 296 with limited cutaneous SSc.^43-46,54,55,58,66,68,70^ Six studies were conducted in Asia,^43,45,54,55,66,68^ and four in Europe.^44,46,58,70^ Endothelin-1 was measured in plasma in 4 studies,^43,46,54,55^ and serum in six.^44,45,58,66,68,70^ An enzyme-linked immunosorbent assay was used in 8 studies,^43,45,54,55,58,66,68,70^ and a radioimmunoassay in the remaining two.^44,46^ Patients received treatment with endothelin receptor antagonists in 2 studies.^58,66^ The risk of bias (Supplemental Table 2) was low in 5 studies,^43,46,55,66,70^ and moderate in the remaining five.^44,45,54,58,68^

**Table 2.** Characteristics of studies investigating endothelin concentrations in patients with systemic sclerosis with limited and diffuse cutaneous SSc.

Study	Matrix	Limited cutaneous SSc				Diffuse cutaneous SSc				
		n	Age (years)	M/F	Endothelin (Mean ± SD)	n	Age (years)	M/F	Endothelin (Mean ± SD)	MDD (years)
Yamane K et al, 1992, Japan^43^	P	13	55.4	1/12	1.53 ± 0.19	18	57.9	2/16	2.16 ± 0.44	5.25
Vancheeswaran R et al, 1994, UK^44^	S	35	NR	NR	4.8 ± 2.4	39	NR	NR	7.9 ± 3.12	15.3
Kadono T et al, 1995, Japan^45^	S	44	NR	NR	20.8 ± 31.5	20	NR	NR	44.2 ± 43.3	NR
Morelli S et al, 1995, Italy^46^	P	8	NR	NR	1.68 ± 0.14	40	NR	NR	1.62 ± 0.39	NR
Kim HS et al, 2010, Korea^54^	P	30	NR	NR	3.2 ± 4.06	30	NR	NR	2.68 ± 2.86	7.3
Pehlivan Y et al, 2011, Turkey^55^	P	12	NR	NR	6.38 ± 6.17	43	NR	NR	6.38 ± 11.2	6.36
Cozzani A et al, 2013, Italy^58^	S	3	NR	NR	5.9 ± 5.26	14	NR	NR	7.27 ± 0.85	NR
Hajialilo M et al, 2017, Iran^66^	S	18	NR	NR	0.62 ± 0.24	42	NR	NR	0.6 ± 0.28	6.62
Nazemiyeh M et al, 2020, Iran^68^	S	23	NR	NR	0.6 ± 0.27	24	NR	NR	0.5 ± 0.18	7.5
Stochmal A et al, 2021, Poland^70^	S	74	NR	NR	1.81 ± 1.04	26	NR	NR	2.03 ± 1.24	8.74

Abbreviations: MDD, mean disease duration; M/F, male to female ratio; NR, not reported; P, plasma; S, serum.

**Figure 5.**
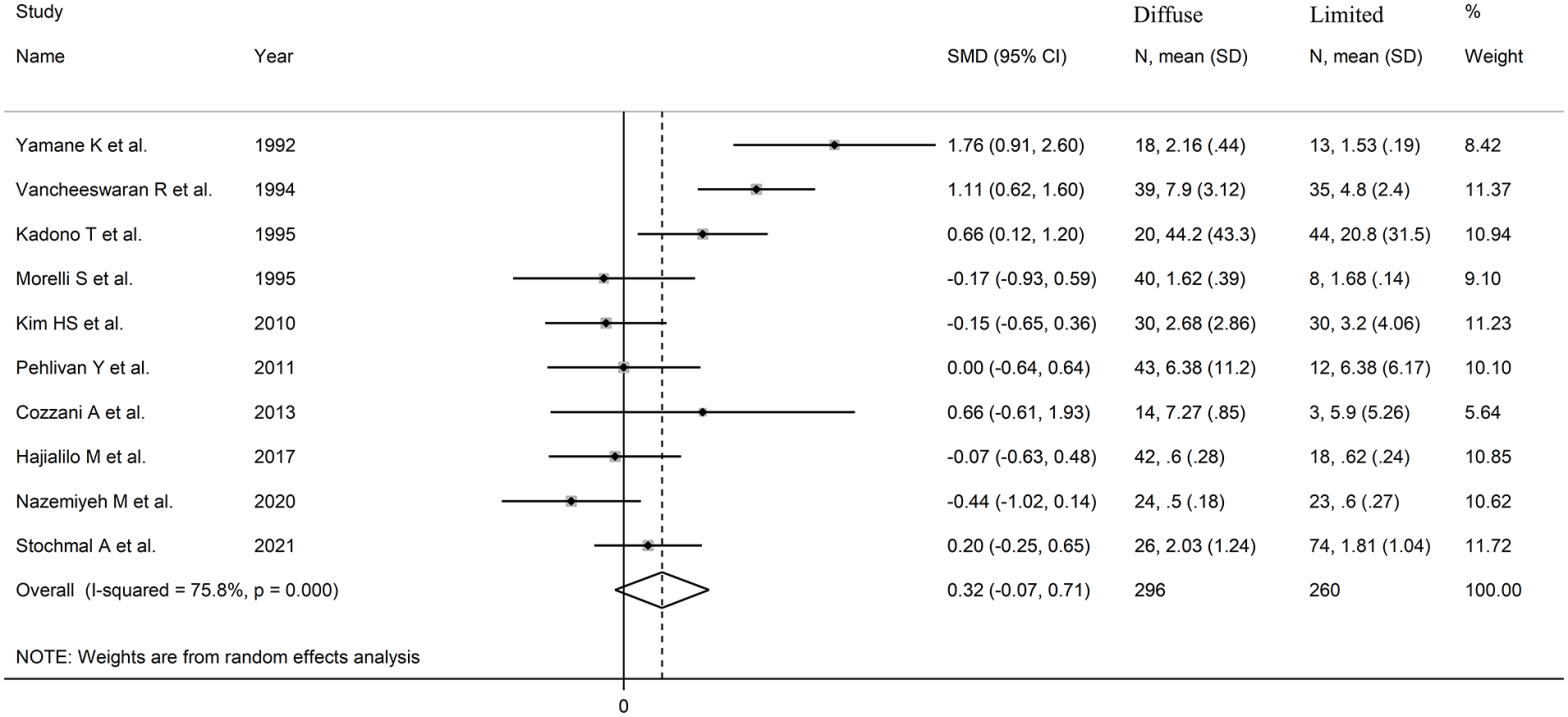
Forest plot of studies on endothelin in patients with limited cutaneous versus diffuse cutaneous SSc.

**Figure 6.**
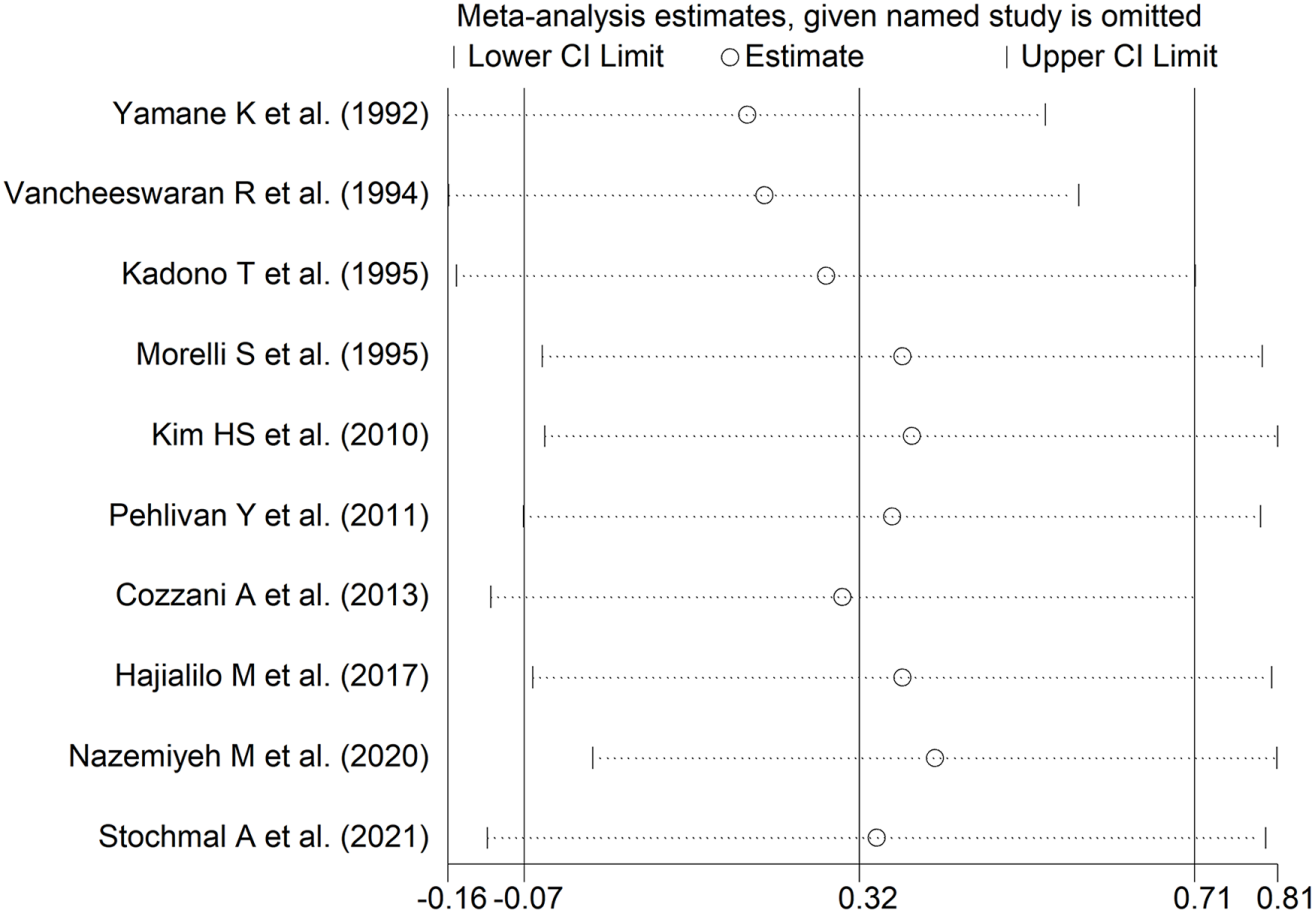
Sensitivity analysis of the association between endothelin and limited cutaneous versus diffuse cutaneous SSc.

The forest plot (Figure 5) showed no significant differences in endothelin concentrations between patients with limited and diffuse cutaneous SSc (SMD = 0.32, 95% CI −0.07 to 0.71 *P* = .11; *I*^2^ = 75.8%, *P*< .001). However, the sensitivity analysis (SMD ranging between 0.19 and 0.41; Figure 6) showed that the effect size was significant (SMD = 0.41; 95% CI 0.41-0.81; *P* = .045, *I*^2^ = 73.8%, *P* < .001) after excluding the study by Nazemiyeh et al.^68^

**Figure 7.**
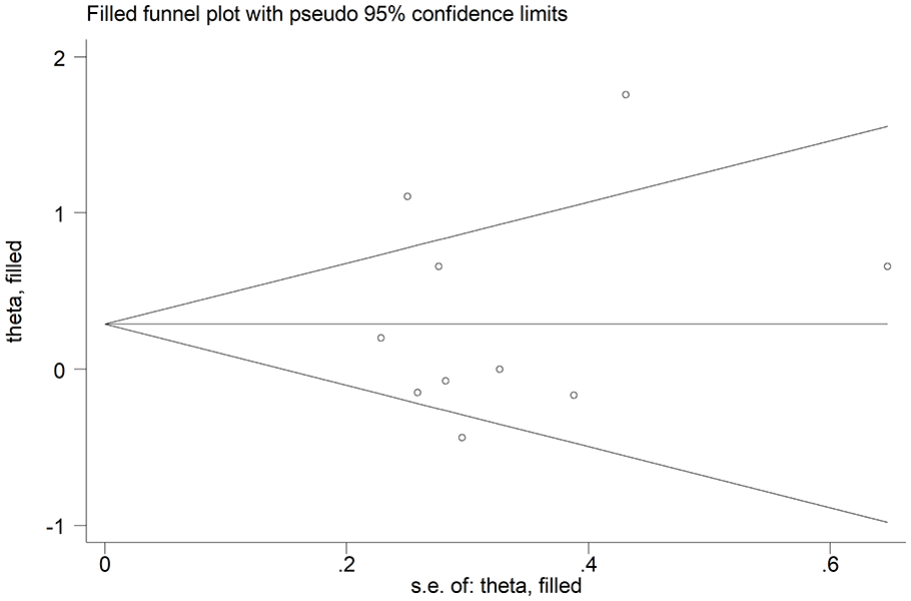
Funnel plot of studies on the association between endothelin and limited cutaneous versus diffuse cutaneous SSc after ‘trimming-and-filling’. Dummy and genuine studies are represented by enclosed circles and free circles, respectively.

We observed no publication bias with Begg’s (*P* = 1.00) and Egger’s test (*P* = .66). Accordingly, the ‘trim-and-fill’ method did not identify any missing study to be added to the funnel plot to ensure symmetry (Figure 7).

In univariate meta-regression analysis, we observed no significant associations between the effect size and the number of participants (*t* = −0.43, *P* = .62). However, there was a significant and inverse association with publication year (*t* = −2.67, *P* = .028; Supplemental Figure 5A) as also confirmed by cumulative analysis performed using the metacum command (Supplemental Figure 5B). In sub-group analysis, we observed no significant differences (*P* = .67) in the pooled SMD between Asian (SMD = 0.24, 95% CI −0.28 to 0.77, *P* = .36; *I*^2^ = 78.7%, *P* < .001) and European studies (SMD = 0.45, 95% CI −0.16 to 1.07, *P* = .15; *I*^2^ = 71.6%, *P* = .014; Supplemental Figure 6). Similarly, we observed no significant differences (*P* = .96) in the pooled SMD between studies assessing plasma (SMD = 0.32, 95% CI −0.46 to 1.10, *P* = .42; *I*^2^ = 81.4%, *P* = .001) and serum (SMD = 0.33, 95% CI −0.15 to 0.81, *P* = .17; *I*^2^ = 75.5%, *P* = .001; Supplemental Figure 7).

We downgraded the final level of the certainty of evidence to very low (level 1) after considering the low-moderate risk of bias in all studies (no change), the high and unexplained heterogeneity (downgrade 1 level), the lack of indirectness (no change), the small effect size (SMD = 0.32, no change),^75^ and the absence of publication bias (no change).

### Endothelin-1 and nailfold video capillaroscopy pattern

One European study with a low risk of bias (Supplemental Table 2) investigated plasma endothelin-1 in 99 SSc patients with different video capillaroscopy pattern (early, n = 27; active, n = 43; late, n = 29).^53^ Endothelin-1 concentrations were significantly lower (*P* = .03) in patients with early pattern [median 1.8 fmol/mL (interquartile range, IQR, 1.9)] when compared with those with late pattern [median 2.6 fmol/mL (IQR 1.8)]. However, there were no significant differences between patients with early and active pattern [median 2.1 fmol/mL (IQR 2.1)] or between active and late pattern.

### Endothelin-1 and digital ulcers

Five studies (Table 3) investigated endothelin-1 in SSc patients with (n = 102) and without (n = 216) digital ulcers.^56,58,64,67,70^ Four studies were conducted in Europe,^58,64,67,70^ and one in Asia.^56^ Two studies measured plasma,^56,64^ and 3 serum.^58,67,70^ An enzyme-linked immunosorbent assay was used in 3 studies,^56,58,70^ a radioimmunoassay in one,^64^ and a platform for multi-analyte profiling in the remaining one.^67^ SSc patients received treatment with endothelin receptor antagonists in one study.^58^ The risk of bias (Supplemental Table 2) was low in 3 studies,^64,67,70^ and moderate in the remaining two.^56,58^

**Table 3.** Characteristics of studies investigating endothelin concentrations in patients with systemic sclerosis with and without complications.

Study	Matrix	Absence of complications				Presence of complications				
		n	Age (years)	M/F	Endothelin (mean ± SD)	n	Age (years)	M/F	Endothelin (mean ± SD)	MDD (years)
*Digital ulcers*										
Aghaei M et al, 2012, Iran^56^	P	78	NR	NR	18.01 ± 5.83	17	NR	NR	39.52 ± 16.89	NR
Cozzani A et al, 2013, Italy^58^	S	11	NR	NR	4.97 ± 1.23	7	NR	NR	7.72 ± 7.31	NR
Silva I et al, 2015, Portugal^64^	P	38	53.2	0/38	9.13 ± 5.23	39	52.7	4/34	16.09 ± 7.86	NR
Nicola S et al, 2017, Italy^67^	S	19	NR	NR	17.3 ± 6.3	9	NR	NR	14.7 ± 0.8	NR
Stochmal A et al, 2021, Poland^70^	S	70	NR	NR	1.74 ± 1.09	30	NR	NR	2.16 ± 1.06	8.74
*Pulmonary arterial hypertension*										
Vancheeswaran R et al, 1994, UK^44^	S	38	NR	NR	5.97 ± 2.84	25	NR	NR	6.7 ± 3	NR
Morelli S et al, 1995, Italy^46^	P	11	48.5	1/10	1.68 ± 0.31	14	45.1	1/13	1.63 ± 0.28	NR
Michaelis T et al, 2012, Brazil^57^	S	28	NR	NR	1.76 ± 1.34	9	NR	NR	1.28 ± 0.78	42.5
Cozzani A et al, 2013, Italy^58^	S	13	NR	NR	6.18 ± 5.46	5	NR	NR	5.68 ± 1.6	NR
Penn et al, 2013, UK^60^	S	27	52.7	NR	0.878 ± 1.438	26	61.2	NR	3.03 ± 5.204	11.6
Kawashiri S et al, 2014, Japan^62^	P	10	NR	NR	2.38 ± 1.07	14	62.4	0/14	3.22 ± 2.06	13
Lemmers JMJ et al, 2023, Netherlands^73^	S	41	59.49	12/29	9 ± 4	40	70.8	10/30	14 ± 25	9.6
*Interstitial lung disease*										
Vancheeswaran R et al, 1994, UK^44^	S	38	NR	NR	5.97 ± 2.84	36	NR	NR	6.13 ± 2.64	NR
Stochmal A et al, 2021, Poland^70^	S	48	NR	NR	1.88 ± 1.23	52	NR	NR	1.86 ± 0.97	8.74
Pulito-Cueto V et al, 2023, Spain^74^	S	25	60.1	10/15	1.14 ± 0.39	21	60.3	8/13	1.37 ± 0.8	11

Abbreviations: MDD, mean disease duration; M/F, male to female ratio; NR, not reported; P, plasma; S, serum.

**Figure 8.**
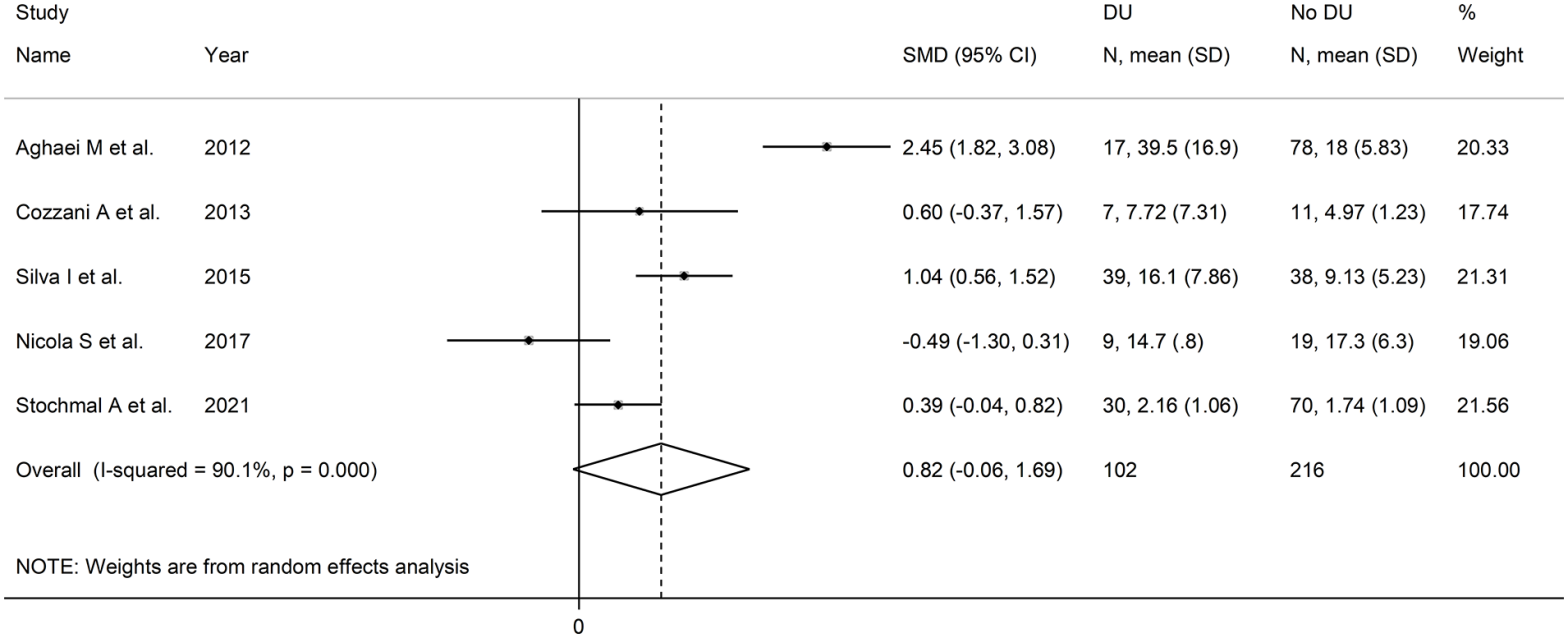
Forest plot of studies assessing endothelin in patients with and without digital ulcers.

**Figure 9.**
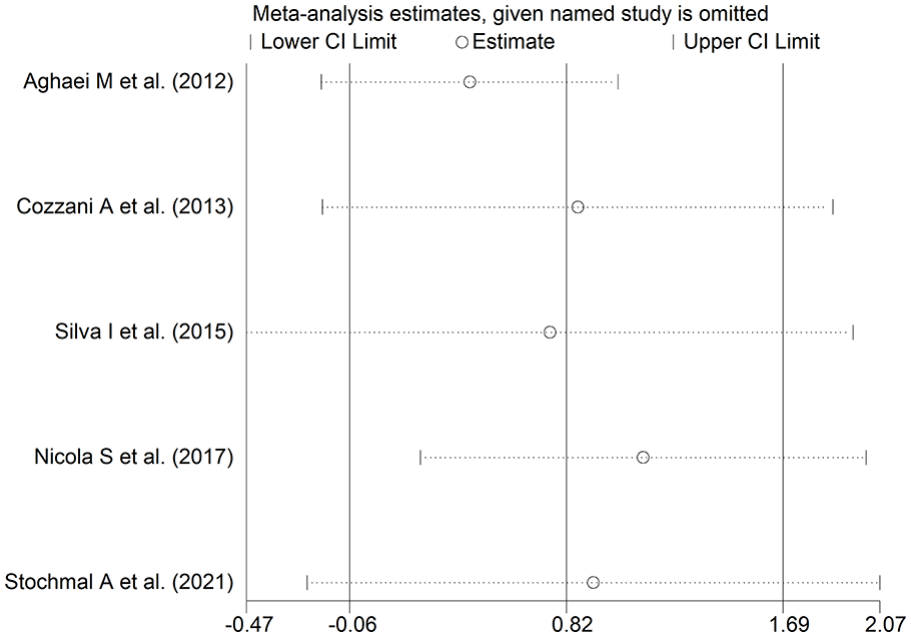
Sensitivity analysis of the association between endothelin and digital ulcers.

The forest plot (Figure 8) showed that patients with digital ulcers had a non-significant trend towards higher endothelin-1 concentrations compared to patients without (SMD = 0.82, 95% CI −0.06 to 1.69, *P* = .066; *I*^2^ = 90.1%, *P* < .001). However, the sensitivity analysis (SMD ranging between 0.43 and 1.12; Figure 9) showed that the effect size was significant (SMD = 1.12, 95% CI 0.23-2.02; *P* = .014, *I*^2^ = 89.5%, *P* < .001) after excluding the study by Nicola et al.^67^

Assessment of publication bias and meta-regression analysis could not be performed because of the small number of studies. In subgroup analysis, the pooled SMD was significantly different in studies measuring plasma (SMD = 1.73, 95% CI 0.35-3.11, *P* = .014; *I*^2^ = 91.8%, *P* < .001) but not serum (SMD = 0.18, 95% CI −0.41 to 0.77, *P* = .55; *I*^2^ = 52.2%, *P* = .12; Supplemental Figure 8), with a lower heterogeneity in the serum subgroup.

The final level of the certainty of evidence was downgraded to very low (level 1) because of the lack of assessment of publication bias.

### Endothelin-1 and pulmonary arterial hypertension

Seven studies (Table 3) measured endothelin-1 in SSc patients with (n = 133) and without (n = 168) pulmonary arterial hypertension.^44,46,57,58,60,62,73^ Five studies were conducted in Europe,^44,46,58,60,73^ one in America,^57^ and one in Asia.^62^ Endothelin-1 was measured in serum in 5 studies,^44,57,58,60,73^ and plasma in two.^46,62^ An enzyme-linked immunosorbent assay was used in 4 studies,^57,58,60,62^ a radioimmunoassay in two,^44,46^ and a platform for multi-analyte profiling in one.^73^ SSc patients received treatment with endothelin receptor antagonists in 3 studies.^58,60,73^ The risk of bias (Supplemental Table 2) was low in 3 studies,^46,57,73^ and moderate in the remaining four.^44,58,60,62^

**Figure 10.**
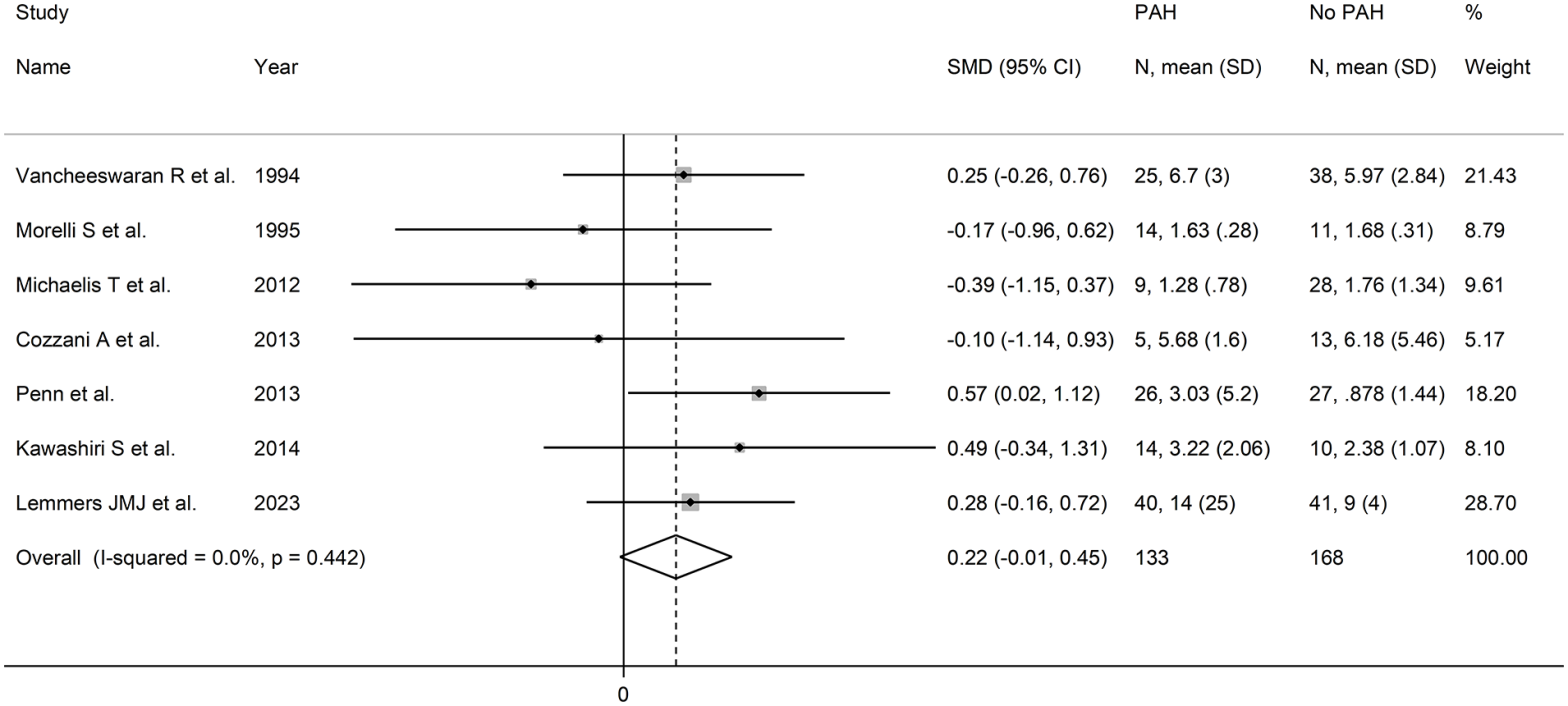
Forest plot of studies on endothelin in patients with and without pulmonary arterial hypertension.

**Figure 11.**
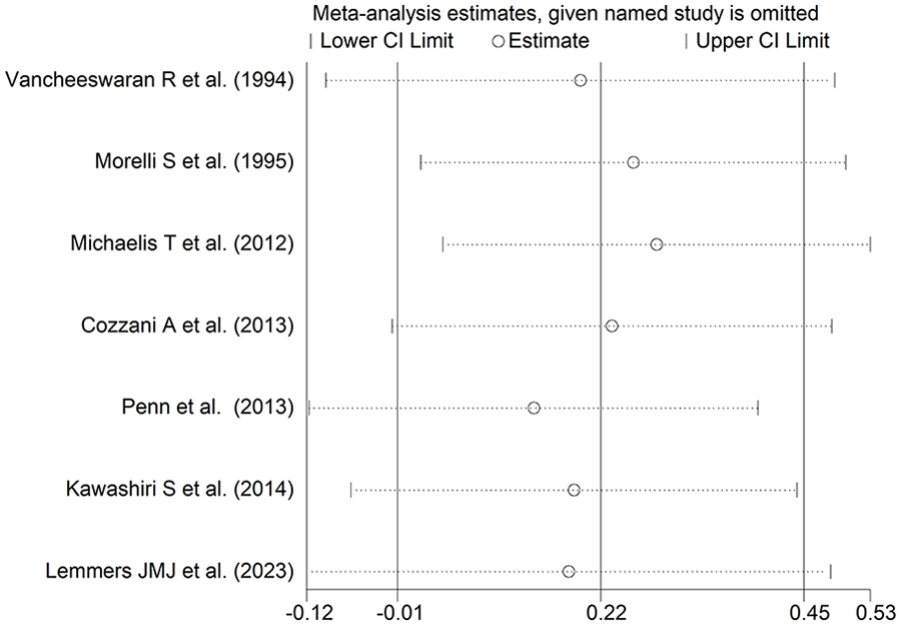
Sensitivity analysis of the association between endothelin and pulmonary arterial hypertension.

The forest plot (Figure 10) showed that SSc patients with pulmonary arterial hypertension had a non-significant trend towards higher endothelin-1 concentrations compared to those without (SMD = 0.22, 95% CI −0.01 to 0.45, *P* = .066; *I*^2^ = 0.0%, *P* = .44). However, the sensitivity analysis (SMD ranging between 0.14 and 0.28; Figure 11) showed that the effect size was significant after excluding the study by Morelli et al,^46^ (SMD = 0.26, 95% CI 0.01-0.50; *P* = .040, *I*^2^ = 0.0%, *P* = .44), or the study by Michaelis et al,^57^ (SMD = 0.28, 95% CI 0.04-0.53; *P* = .028, *I*^2^ = 0.0%, *P* = .69).

Assessment of publication bias and meta-regression could not be performed because of the small number of studies. In subgroup analysis, we observed no significant differences (*P* = .80) in the pooled SMD between studies measuring serum (SMD = 0.23, 95% CI −0.05 to 0.50, *P* = .11; *I*^2^ = 10.9%, *P* = .34) and plasma (SMD = 0.15, 95% CI −0.50 to 0.79, *P* = .65; *I*^2^ = 21.4%, *P* = .26; Supplemental Figure 9). Similarly, we observed no significant differences (*P* = .78) in the pooled SMD between studies using an enzyme-linked immunosorbent assay (SMD = 0.20, 95% CI −0.28 to 0.68, *P* = .42; *I*^2^ = 37.5%, *P* = .19) and a radioimmunoassay (SMD = 0.13, 95% CI −0.30 to 0.56, *P* = .55; *I*^2^ = 0.0%, *P* = .38; Supplemental Figure 10), with a virtually absent heterogeneity in the radioimmunoassay subgroup.

The final level of the certainty of evidence was downgraded to very low (level 1) because of the lack of assessment of publication bias.

### Endothelin-1 and interstitial lung disease

Three European studies (Table 3) investigated serum endothelin-1 in 109 SSc patients with interstitial lung disease and 111 without.^44,70,74^ An enzyme-linked immunosorbent assay was used in 2 studies,^70,74^ and a radioimmunoassay in the remaining one.^44^ The risk of bias (Supplemental Table 2) was low in one study,^70^ and moderate in the other two.^44,74^

**Figure 12.**
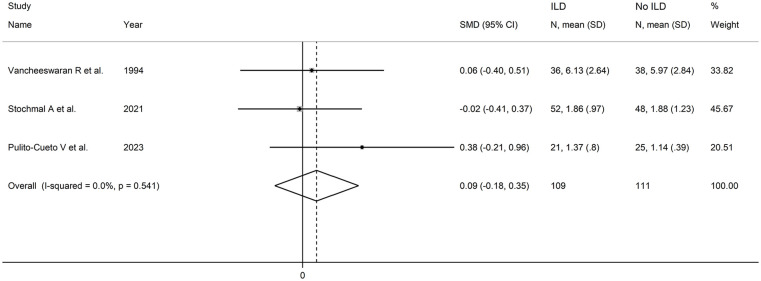
Forest plot of studies investigating endothelin in patients with and without interstitial lung disease.

The forest plot (Figure 12) showed no significant between-group differences in endothelin-1 concentrations (SMD = 0.09, 95% CI −0.18 to 0.35, *P* = .51, *I*^2^ = 0.0%, *P* = .54). Assessment of sensitivity, publication bias, meta-regression and sub-group analysis could not be performed because of the small number of studies. Consequently, the final level of the certainty of evidence was downgraded to very low (level 1).

### Endothelin-1 and telangiectasias

One American study with a low risk of bias (Supplemental Table 2) investigated serum endothelin-1 in 18 SSc patients with telangiectasias and 19 without.^57^ No significant between-group differences were observed (median: 0.41 pg/mL, IQR 0.41 to 0.82 pg/mL vs 0.62 pg/mL, IQR 0.41-2.79 pg/mL, *P* = .74).

## Discussion

This systematic review and meta-analysis has shown that, overall, patients with SSc have significant elevations in endothelin-1 concentrations when compared to healthy controls. No significant differences in circulating endothelin-1 were observed between specific SSc subgroups, that is, patients with limited versus diffuse cutaneous SSc and with versus without interstitial lung disease. A trend towards a significant increase in endothelin-1 concentrations was observed in SSc patients with versus without digital ulcers and pulmonary arterial hypertension. There was limited evidence regarding endothelin-1 concentrations in SSc patients with different video capillaroscopy pattern and telangiectasias. Meta-regression and subgroup analyses showed no significant associations between the effect size of the between-group differences in endothelin-1 concentrations and various patient and study characteristics, except for geographical location (studies investigating SSc patients and controls), year of publication (studies investigating SSc patients with limited or diffuse cutaneous SSc) and biological matrix assessed (studies investigating SSc patients with and without digital ulcers). The results of the meta-analysis were stable in sensitivity analysis. Taken together, our results highlight the potential role of endothelin-1 as a candidate biomarker of SSc although further research is required to determine the utility of measuring this peptide in specific subsets of SSc patients. Notably, the absence of significant associations between the magnitude of the elevations in endothelin-1 in SSc patients and disease duration indicates that such elevations are already present in the early phases of the disease.^9^

The vasoconstrictive, pro-angiogenic, and pro-fibrotic effects of endothelin-1 have been investigated in several disease states.^13,16,18-28^ Such effects play a critical role in the pathogenesis and progression of SSc.^8,10-12^ The pro-fibrotic effects of endothelin-1 have been particularly well studied in SSc in the context of a dysregulation of the TGF-β/endothelin-1/Ras signalling pathway.^27,28^ TGF-β stimulates the release of endothelin-1 not only from endothelial cells but also from fibroblasts and myofibroblasts. This, in turn, causes increased blood vessel permeability, altered vascular tone, hypoxia, endothelial damage, morphological microvascular abnormalities, and excess fibrosis.^76,77^ The TGF-β/endothelin-1/Ras signalling pathway has been shown to protect myofibroblasts from apoptosis, further perpetuating the pro-fibrotic effect.^28,78^ Additional pro-fibrotic effects of endothelin-1 are mediated by the upregulation of connective tissue growth factor (CTGF),^79^ and the stimulation of endothelial-to-mesenchymal transition.^80^ The pathophysiological relevance of endothelin-1 in SSc is further supported by the efficacy of endothelin receptor antagonists in managing specific SSc patient subgroups, for example, those with digital ulcers and pulmonary arterial hypertension.^31,81-83^ The observed trends, in our study, towards significant elevations in endothelin-1 in SSc patients with digital ulcers or pulmonary arterial hypertension warrant further studies to support the utility of measuring endothelin-1 in these subgroups.

Another interesting observation in our subgroup analysis of studies investigating SSc patients and healthy controls was that significant elevations in endothelin-1 were observed in European and Asian studies but not in studies conducted in the American region. There is good evidence of geographical variations in the clinical manifestations of SSc. A systematic review and meta-analysis has shown that people residing in Asia have an earlier disease onset, a lower prevalence of telangiectasias and a higher prevalence of pulmonary involvement compared to subjects residing in Europe, North America, and South America.^84^ Other studies have reported an earlier disease onset, greater pulmonary involvement and higher risk of mortality in Asian patients and black patients when compared to white patients.^85^ However, no significant ethnic-related differences in survival have been reported in other studies.^86^ Studies have also highlighted the presence of ethnic-related differences in endothelin-1 concentrations, with higher concentrations observed in black subjects compared to white individuals.^87,88^ Further research is therefore required to investigate the potential influence of ethnicity and geographical location on the alterations in endothelin-1 concentrations in SSc patients overall and in specific subgroups.

Another factor potentially influencing the results of our systematic review and meta-analysis is the effect of pharmacological treatment on endothelin-1 concentrations. Five of the studies identified in our meta-analysis included SSc patients receiving treatment with endothelin receptor antagonists.^58,60,66,71,73^ There is good evidence that endothelin receptor antagonists can acutely increase endothelin-1 concentrations in healthy subjects and different patient groups, including those with pulmonary hypertension.^89,90^ However, chronic treatment with vasodilators, including endothelin receptor antagonists, can significantly lower circulating endothelin-1 in pulmonary arterial hypertension, indicating treatment response.^91,92^ Therefore, future studies investigating endothelin-1 as candidate biomarker in SSc should also take into account the potential influencing role of different therapies and their duration. Such studies should also investigate whether circulating endothelin-1 concentrations correlate with the levels of the peptide in organs and tissues primarily affected in SSc, for example, skin, blood vessels and lung parenchyma.^93,94^

Strengths of our study include the evaluation of endothelin-1 in a wide range of SSc patient subtypes and the assessment of the certainty of evidence for each meta-analysed endpoint. Significant limitations include the relatively small number of studies investigating endothelin-1 in SSc patients with and without digital ulcers, pulmonary arterial hypertension, interstitial lung disease, telangiectasias and different capillaroscopy pattern. This prevented conducting meta-regression and subgroup analyses to identify associations between the effect size and various study and patient characteristics as well as sources of heterogeneity.

## Conclusions

We observed significant elevations in circulating endothelin-1 in patients with SSc. Further, accurately designed, prospective studies are warranted to investigate the diagnostic as well as the prognostic capacity of this candidate biomarker in SSc patients with different clinical manifestations (eg, limited vs diffuse cutaneous SSc, different nailfold video capillaroscopy patterns, and presence vs absence of digital ulcers, telangiectasias, pulmonary arterial hypertension, or interstitial lung disease). Such studies should also investigate the role of ethnicity and pharmacological treatments to comprehensively determine the potential clinical utility of measuring endothelin-1 in the assessment and monitoring of SSc patients. The results of these studies might also allow the identification of novel pathophysiological pathways and therapeutic strategies to enhance health outcomes in this group.

## List of Abbreviations

CI: Confidence interval.

CTGF: Connective tissue growth factor.

ET_A_: Endothelin A receptor.

ET_B_: Endothelin B receptor.

GRADE: Grades of Recommendation, Assessment, Development, and Evaluation.

HIF-1: Heterodimer hypoxia-inducible factor-1.

IQR: Interquartile range.

JBI: Johanna Briggs Institute.

PROSPERO: International Prospective Register of Systematic Reviews.

SMD: Standardised mean difference.

SSc: systemic sclerosis.

TGF-β: Transforming growth factor β.

VEGF: Vascular endothelial growth factor.

## Supplemental Material

sj-docx-1-bmi-10.1177_11772719251318555 – Supplemental material for Endothelin-1 as a Candidate Biomarker of Systemic Sclerosis: A GRADE-Assessed Systematic Review and Meta-Analysis With Meta-Regression Supplemental material, sj-docx-1-bmi-10.1177_11772719251318555 for Endothelin-1 as a Candidate Biomarker of Systemic Sclerosis: A GRADE-Assessed Systematic Review and Meta-Analysis With Meta-Regression by Arduino A Mangoni and Angelo Zinellu in Biomarker Insights

sj-docx-2-bmi-10.1177_11772719251318555 – Supplemental material for Endothelin-1 as a Candidate Biomarker of Systemic Sclerosis: A GRADE-Assessed Systematic Review and Meta-Analysis With Meta-Regression Supplemental material, sj-docx-2-bmi-10.1177_11772719251318555 for Endothelin-1 as a Candidate Biomarker of Systemic Sclerosis: A GRADE-Assessed Systematic Review and Meta-Analysis With Meta-Regression by Arduino A Mangoni and Angelo Zinellu in Biomarker Insights

sj-docx-3-bmi-10.1177_11772719251318555 – Supplemental material for Endothelin-1 as a Candidate Biomarker of Systemic Sclerosis: A GRADE-Assessed Systematic Review and Meta-Analysis With Meta-Regression Supplemental material, sj-docx-3-bmi-10.1177_11772719251318555 for Endothelin-1 as a Candidate Biomarker of Systemic Sclerosis: A GRADE-Assessed Systematic Review and Meta-Analysis With Meta-Regression by Arduino A Mangoni and Angelo Zinellu in Biomarker Insights

sj-tif-10-bmi-10.1177_11772719251318555 – Supplemental material for Endothelin-1 as a Candidate Biomarker of Systemic Sclerosis: A GRADE-Assessed Systematic Review and Meta-Analysis With Meta-Regression Supplemental material, sj-tif-10-bmi-10.1177_11772719251318555 for Endothelin-1 as a Candidate Biomarker of Systemic Sclerosis: A GRADE-Assessed Systematic Review and Meta-Analysis With Meta-Regression by Arduino A Mangoni and Angelo Zinellu in Biomarker Insights

sj-tif-11-bmi-10.1177_11772719251318555 – Supplemental material for Endothelin-1 as a Candidate Biomarker of Systemic Sclerosis: A GRADE-Assessed Systematic Review and Meta-Analysis With Meta-Regression Supplemental material, sj-tif-11-bmi-10.1177_11772719251318555 for Endothelin-1 as a Candidate Biomarker of Systemic Sclerosis: A GRADE-Assessed Systematic Review and Meta-Analysis With Meta-Regression by Arduino A Mangoni and Angelo Zinellu in Biomarker Insights

sj-tif-12-bmi-10.1177_11772719251318555 – Supplemental material for Endothelin-1 as a Candidate Biomarker of Systemic Sclerosis: A GRADE-Assessed Systematic Review and Meta-Analysis With Meta-Regression Supplemental material, sj-tif-12-bmi-10.1177_11772719251318555 for Endothelin-1 as a Candidate Biomarker of Systemic Sclerosis: A GRADE-Assessed Systematic Review and Meta-Analysis With Meta-Regression by Arduino A Mangoni and Angelo Zinellu in Biomarker Insights

sj-tif-13-bmi-10.1177_11772719251318555 – Supplemental material for Endothelin-1 as a Candidate Biomarker of Systemic Sclerosis: A GRADE-Assessed Systematic Review and Meta-Analysis With Meta-Regression Supplemental material, sj-tif-13-bmi-10.1177_11772719251318555 for Endothelin-1 as a Candidate Biomarker of Systemic Sclerosis: A GRADE-Assessed Systematic Review and Meta-Analysis With Meta-Regression by Arduino A Mangoni and Angelo Zinellu in Biomarker Insights

sj-tif-4-bmi-10.1177_11772719251318555 – Supplemental material for Endothelin-1 as a Candidate Biomarker of Systemic Sclerosis: A GRADE-Assessed Systematic Review and Meta-Analysis With Meta-Regression Supplemental material, sj-tif-4-bmi-10.1177_11772719251318555 for Endothelin-1 as a Candidate Biomarker of Systemic Sclerosis: A GRADE-Assessed Systematic Review and Meta-Analysis With Meta-Regression by Arduino A Mangoni and Angelo Zinellu in Biomarker Insights

sj-tif-5-bmi-10.1177_11772719251318555 – Supplemental material for Endothelin-1 as a Candidate Biomarker of Systemic Sclerosis: A GRADE-Assessed Systematic Review and Meta-Analysis With Meta-Regression Supplemental material, sj-tif-5-bmi-10.1177_11772719251318555 for Endothelin-1 as a Candidate Biomarker of Systemic Sclerosis: A GRADE-Assessed Systematic Review and Meta-Analysis With Meta-Regression by Arduino A Mangoni and Angelo Zinellu in Biomarker Insights

sj-tif-6-bmi-10.1177_11772719251318555 – Supplemental material for Endothelin-1 as a Candidate Biomarker of Systemic Sclerosis: A GRADE-Assessed Systematic Review and Meta-Analysis With Meta-Regression Supplemental material, sj-tif-6-bmi-10.1177_11772719251318555 for Endothelin-1 as a Candidate Biomarker of Systemic Sclerosis: A GRADE-Assessed Systematic Review and Meta-Analysis With Meta-Regression by Arduino A Mangoni and Angelo Zinellu in Biomarker Insights

sj-tif-7-bmi-10.1177_11772719251318555 – Supplemental material for Endothelin-1 as a Candidate Biomarker of Systemic Sclerosis: A GRADE-Assessed Systematic Review and Meta-Analysis With Meta-Regression Supplemental material, sj-tif-7-bmi-10.1177_11772719251318555 for Endothelin-1 as a Candidate Biomarker of Systemic Sclerosis: A GRADE-Assessed Systematic Review and Meta-Analysis With Meta-Regression by Arduino A Mangoni and Angelo Zinellu in Biomarker Insights

sj-tif-8-bmi-10.1177_11772719251318555 – Supplemental material for Endothelin-1 as a Candidate Biomarker of Systemic Sclerosis: A GRADE-Assessed Systematic Review and Meta-Analysis With Meta-Regression Supplemental material, sj-tif-8-bmi-10.1177_11772719251318555 for Endothelin-1 as a Candidate Biomarker of Systemic Sclerosis: A GRADE-Assessed Systematic Review and Meta-Analysis With Meta-Regression by Arduino A Mangoni and Angelo Zinellu in Biomarker Insights

sj-tif-9-bmi-10.1177_11772719251318555 – Supplemental material for Endothelin-1 as a Candidate Biomarker of Systemic Sclerosis: A GRADE-Assessed Systematic Review and Meta-Analysis With Meta-Regression Supplemental material, sj-tif-9-bmi-10.1177_11772719251318555 for Endothelin-1 as a Candidate Biomarker of Systemic Sclerosis: A GRADE-Assessed Systematic Review and Meta-Analysis With Meta-Regression by Arduino A Mangoni and Angelo Zinellu in Biomarker Insights

## Declarations

**Ethics approval and consent to participate:** Not applicable.

**Consent for publication:** Not applicable.

**Availability of data and materials:** The data that support the findings of this systematic review and meta-analysis are available from AZ upon reasonable request.
