# Supplementary material for: Endothelin-1 as a Candidate Biomarker of Systemic Sclerosis: A GRADE-Assessed Systematic Review and Meta-Analysis With Meta-Regression
Source: Biomark Insights. 2025 Feb 21;20:11772719251318555. doi: 10.1177/11772719251318555 (PMC11846126; doi:10.1177/11772719251318555)
Supplement: sj-docx-1-bmi-10.1177_11772719251318555 – Supplemental material for Endothelin-1 as a Candidate Biomarker of Systemic Sclerosis: A GRADE-Assessed Systematic Review and Meta-Analysis With Meta-Regression [file sj-docx-1-bmi-10.1177_11772719251318555.docx]

**Supplementary figure legends**

**Supplementary Figure 1.** Forest plot of studies investigating endothelin in patients with systemic sclerosis and healthy controls according to geographical location.

**Supplementary Figure 2.** Forest plot of studies investigating endothelin in patients with systemic sclerosis and healthy controls according to biological matrix assessed.

**Supplementary Figure 3.** Forest plot of studies investigating endothelin in patients with systemic sclerosis and healthy controls according to analytical method.

**Supplementary Figure 4.** Forest plot of studies investigating endothelin in patients with systemic sclerosis and healthy controls according to biological matrix and analytical method.

**Supplementary Figure 5.** Bubble plot reporting univariate meta-regression analysis between the effect size and publication year (A) and cumulative meta-analysis of endothelin concentrations based on the year of publication (B).

**Supplementary Figure 6.** Forest plot of studies investigating endothelin in patients with systemic sclerosis with localized and diffuse disease according to geographical location.

**Supplementary Figure 7.** Forest plot of studies investigating endothelin in patients with systemic sclerosis with localized and diffuse disease according to biological matrix assessed.

**Supplementary Figure 8.** Forest plot of studies investigating endothelin in patients with systemic sclerosis with and without digital ulcers according to biological matrix assessed.

**Supplementary Figure 9.** Forest plot of studies investigating endothelin in patients with systemic sclerosis with and without pulmonary arterial hypertension according to biological sample matrix assessed.

**Supplementary Figure 10.** Forest plot of studies endothelin in patients with systemic sclerosis with and without pulmonary arterial hypertension according to analytical method.
