# Supplementary material for: Endothelin-1 as a Candidate Biomarker of Systemic Sclerosis: A GRADE-Assessed Systematic Review and Meta-Analysis With Meta-Regression
Source: Biomark Insights. 2025 Feb 21;20:11772719251318555. doi: 10.1177/11772719251318555 (PMC11846126; doi:10.1177/11772719251318555)
Supplement: sj-docx-3-bmi-10.1177_11772719251318555 – Supplemental material for Endothelin-1 as a Candidate Biomarker of Systemic Sclerosis: A GRADE-Assessed Systematic Review and Meta-Analysis With Meta-Regression [file sj-docx-3-bmi-10.1177_11772719251318555.docx]

**Supplementary Table 2.** Assessment of the risk of bias using the Joanna Briggs Institute critical appraisal checklist.

| **Study** | **Were the inclusion criteria clearly defined?** | **Were the subjects and the setting described in detail?** | **Was the exposure measured in a reliable way?** | **Were standard criteria used to assess the condition?** | **Were confounding factors identified?** | **Were strategies to deal with confounding factors stated?** | **Were the outcomes measured in a reliable way?** | **Was appropriate statistical analysis used?** | **Risk of bias** |
| --- | --- | --- | --- | --- | --- | --- | --- | --- | --- |
| Yamane K et al. ^1^ | No | Yes | Yes | Yes | Yes | Yes | Yes | Yes | Low |
| Vancheeswaran R et al. ^2^ | No | Yes | Yes | Yes | No | No | Yes | Yes | Moderate |
| Kadono T et al. ^3^ | No | Yes | Yes | Yes | No | No | Yes | Yes | Moderate |
| Morelli S et al. ^4^ | Yes | Yes | Yes | Yes | Yes | Yes | Yes | Yes | Low |
| Maeda M et al. ^5^ | No | Yes | Yes | Yes | No | No | Yes | Yes | Moderate |
| Silveri F et al. ^6^ | Yes | Yes | Yes | Yes | No | No | Yes | Yes | Low |
| Fontana F et al. ^7^ | Yes | Yes | Yes | No | No | No | Yes | Yes | Moderate |
| Kuryliszyn-Moskal A et al. ^8^ | No | Yes | Yes | Yes | No | No | Yes | Yes | Moderate |
| Peterlana D et al. ^9^ | Yes | Yes | Yes | No | No | No | Yes | Yes | Moderate |
| Coral-Alvarado P et al. ^10^ | Yes | Yes | Yes | Yes | No | No | Yes | Yes | Low |
| Sulli A et al. ^11^ | Yes | Yes | Yes | Yes | No | No | Yes | Yes | Low |
| Kim HS et al. ^12^ | No | Yes | Yes | Yes | No | No | Yes | Yes | Moderate |
| Pehlivan Y et al. ^13^ | Yes | Yes | Yes | Yes | No | No | Yes | Yes | Low |
| Aghaei M et al. ^14^ | No | No | Yes | Yes | No | No | Yes | Yes | Moderate |
| Michaelis T et al. ^15^ | Yes | Yes | Yes | Yes | No | No | Yes | Yes | Low |
| Cozzani A et al. ^16^ | No | Yes | Yes | Yes | No | No | Yes | Yes | Moderate |
| Irzyk K et al. ^17^ | Yes | Yes | Yes | Yes | No | No | Yes | Yes | Low |
| Penn et al. ^18^ | No | Yes | Yes | Yes | No | No | Yes | Yes | Moderate |
| Kawashiri S et al. ^19^ | No | Yes | Yes | Yes | No | No | Yes | Yes | Moderate |
| Yilmaz N et al. ^20^ | Yes | Yes | Yes | Yes | No | No | Yes | Yes | Low |
| Camargo CZ et al. ^21^ | Yes | Yes | Yes | Yes | No | No | Yes | Yes | Low |
| Silva I et al. ^22^ | Yes | Yes | Yes | Yes | No | No | Yes | Yes | Low |
| Benyamine et al. ^23^ | No | Yes | Yes | Yes | Yes | Yes | Yes | Yes | Low |
| Hajialilo M et al. ^24^ | Yes | Yes | Yes | Yes | No | No | Yes | Yes | Low |
| Nicola S et al. ^25^ | Yes | Yes | Yes | Yes | No | No | Yes | Yes | Low |
| Nazemiyeh M et al. ^26^ | Yes | No | Yes | No | No | No | Yes | Yes | Moderate |
| Al-Omary Obadeh M et al. ^27^ | Yes | Yes | Yes | Yes | No | No | Yes | Yes | Low |
| Stochmal A et al. ^28^ | Yes | Yes | Yes | Yes | No | No | Yes | Yes | Low |
| Apti Sengun O et al. ^29^ | Yes | Yes | Yes | Yes | No | No | Yes | Yes | Low |
| Bhattacharjee D et al. ^30^ | Yes | Yes | Yes | Yes | No | No | Yes | Yes | Low |
| Lemmers JMJ et al. ^31^ | Yes | Yes | Yes | Yes | Yes | Yes | Yes | Yes | Low |
| Pulito-Cueto V et al. ^32^ | No | Yes | Yes | Yes | No | No | Yes | Yes | Moderate |

**References**

1. Yamane K, Miyauchi T, Suzuki N, et al. Significance of plasma endothelin-1 levels in patients with systemic sclerosis. *J Rheumatol* 1992; 19: 1566-1571.

2. Vancheeswaran R, Magoulas T, Efrat G, et al. Circulating endothelin-1 levels in systemic sclerosis subsets--a marker of fibrosis or vascular dysfunction? *J Rheumatol* 1994; 21: 1838-1844.

3. Kadono T, Kikuchi K, Sato S, et al. Elevated plasma endothelin levels in systemic sclerosis. *Arch Dermatol Res* 1995; 287: 439-442. DOI: 10.1007/BF00373425.

4. Morelli S, Ferri C, Di Francesco L, et al. Plasma endothelin-1 levels in patients with systemic sclerosis: influence of pulmonary or systemic arterial hypertension. *Ann Rheum Dis* 1995; 54: 730-734. DOI: 10.1136/ard.54.9.730.

5. Maeda M, Kachi H, Takagi H, et al. Is there circadian variation of plasma endothelin (ET-1) in patients with systemic scleroderma (SSc)? *J Dermatol Sci* 1997; 16: 38-44. DOI: 10.1016/s0923-1811(97)00619-1.

6. Silveri F, De Angelis R, Poggi A, et al. Relative roles of endothelial cell damage and platelet activation in primary Raynaud's phenomenon (RP) and RP secondary to systemic sclerosis. *Scand J Rheumatol* 2001; 30: 290-296. DOI: 10.1080/030097401753180372.

7. Fontana F, Bernardi P, Lanfranchi G, et al. Endothelin-1 response to mental stress in early ischemic lesions of the extremities due to systemic sclerosis. *Peptides* 2005; 26: 2487-2490. DOI: 10.1016/j.peptides.2005.06.001.

8. Kuryliszyn-Moskal A, Klimiuk PA and Sierakowski S. Soluble adhesion molecules (sVCAM-1, sE-selectin), vascular endothelial growth factor (VEGF) and endothelin-1 in patients with systemic sclerosis: relationship to organ systemic involvement. *Clin Rheumatol* 2005; 24: 111-116. 20040903. DOI: 10.1007/s10067-004-0987-3.

9. Peterlana D, Puccetti A, Caramaschi P, et al. Endothelin‐1 serum levels correlate with MCP‐1 but not with homocysteine plasma concentration in patients with systemic sclerosis. *Scandinavian Journal of Rheumatology* 2009; 35: 133-137. DOI: 10.1080/03009740500385584.

10. Coral-Alvarado P, Quintana G, Garces MF, et al. Potential biomarkers for detecting pulmonary arterial hypertension in patients with systemic sclerosis. *Rheumatol Int* 2009; 29: 1017-1024. 20081231. DOI: 10.1007/s00296-008-0829-8.

11. Sulli A, Soldano S, Pizzorni C, et al. Raynaud's Phenomenon and Plasma Endothelin: Correlations with Capillaroscopic Patterns in Systemic Sclerosis. *Journal of Rheumatology* 2009; 36: 1235-1239. DOI: 10.3899/jrheum.081030.

12. Kim HS, Park MK, Kim HY, et al. Capillary dimension measured by computer-based digitalized image correlated with plasma endothelin-1 levels in patients with systemic sclerosis. *Clin Rheumatol* 2010; 29: 247-254. 20091231. DOI: 10.1007/s10067-009-1288-7.

13. Pehlivan Y, Onat AM, Comez G, et al. Urotensin-II in systemic sclerosis: a new peptide in pathogenesis. *Clin Rheumatol* 2011; 30: 837-842. 20110128. DOI: 10.1007/s10067-011-1688-3.

14. Aghaei M, Gharibdost F, Zayeni H, et al. Endothelin-1 in systemic sclerosis. *Indian Dermatol Online J* 2012; 3: 14-16. DOI: 10.4103/2229-5178.93484.

15. Michaelis T, Andretta M, Albers C, et al. Evaluation of the capillaroscopy using endothelin-1 as a marker of endothelial activation in microvascular injury and cutaneous ulcerations. *Rev Col Bras Cir* 2012; 39: 126-132.

16. Cozzani E, Javor S, Laborai E, et al. Endothelin-1 levels in scleroderma patients: a pilot study. *ISRN Dermatol* 2013; 2013: 125632. DOI: 10.1155/2013/125632.

17. Irzyk K, Bienias P, Kostrubiec M, et al. Six-minute walk test reflects neurohormonal activation and right ventricular function in systemic sclerosis patients. *Clin Exp Rheumatol* 2013; 31: 18-23. 20130213.

18. Penn H, Quillinan N, Khan K, et al. Targeting the endothelin axis in scleroderma renal crisis: rationale and feasibility. *QJM* 2013; 106: 839-848. 20130521. DOI: 10.1093/qjmed/hct111.

19. Kawashiri SY, Ueki Y, Terada K, et al. Improvement of plasma endothelin-1 and nitric oxide in patients with systemic sclerosis by bosentan therapy. *Rheumatol Int* 2014; 34: 221-225. 20130927. DOI: 10.1007/s00296-013-2861-6.

20. Yilmaz N, Olgun S, Ahiskali R, et al. Decreased sputum caveolin-1 is associated with systemic sclerosis related lung disease. *Sarcoidosis Vasc Diffuse Lung Dis* 2014; 31: 55-61. 20140418. DOI: 10.1007/s11083-013-9288-2.

21. Camargo CZ, Sekiyama JY, Arismendi MI, et al. Microvascular abnormalities in patients with early systemic sclerosis: less severe morphological changes than in patients with definite disease. *Scandinavian Journal of Rheumatology* 2014; 44: 48-55. DOI: 10.3109/03009742.2014.926566.

22. Silva I, Teixeira A, Oliveira J, et al. Predictive value of vascular disease biomarkers for digital ulcers in systemic sclerosis patients. *Clin Exp Rheumatol* 2015; 33: S127-130. 20150805.

23. Benyamine A, Magalon J, Cointe S, et al. Increased serum levels of fractalkine and mobilisation of CD34+ CD45- endothelial progenitor cells in systemic sclerosis. *Arthritis Research & Therapy* 2017; 19. DOI: 10.1186/s13075-017-1271-7.

24. Hajialilo M, Noorabadi P, Tekantapeh S, et al. Endothelin-1, α-Klotho, 25(OH) Vit D levels and severity of disease in scleroderma patients. *Rheumatology International* 2017; 37: 1651-1657. DOI: 10.1007/s00296-017-3797-z.

25. Nicola S, Fornero M, Fusaro E, et al. Th1-and Th17-Related Cytokines in Venous and Arterial Blood of Sclerodermic Patients with and without Digital Ulcers. *Biomed Research International* 2019; 2019: 1-5. DOI: 10.1155/2019/7908793.

26. Nazemiyeh M, Hajalilou M, Rajabnia M, et al. Diagnostic value of Endothelin 1 as a marker for diagnosis of pulmonary parenchyma involvement in patients with systemic sclerosis. *Aims Medical Science* 2020; 7: 234-242. DOI: 10.3934/medsci.2020014.

27. Al-Omary Obadeh M and Bondar S. Endothelial Dysfunction and Pathogenetic Phenotypes of Localized Scleroderma. *Georgian Med News* 2021: 102-108.

28. Stochmal A, Czuwara J, Zaremba M, et al. Metabolic mediators determine the association of antinuclear antibody subtypes with specific clinical symptoms in systemic sclerosis. *Adv Med Sci* 2021; 66: 119-127. 20210122. DOI: 10.1016/j.advms.2020.12.007.

29. Apti Sengun O, Ergun T, Guctekin T, et al. Endothelial dysfunction, thrombophilia, and nailfold capillaroscopic features in livedoid vasculopathy. *Microvascular Research* 2023; 150. DOI: 10.1016/j.mvr.2023.104591.

30. Bhattacharjee D, Mondal S, Saha A, et al. Effect of vasodilator and immunosuppressive therapy on the endothelial dysfunction in patients with systemic sclerosis. *Clin Exp Med* 2023; 23: 905-915. 20220625. DOI: 10.1007/s10238-022-00845-w.

31. Lemmers JM, van Caam AP, Kersten B, et al. Nailfold capillaroscopy and candidate-biomarker levels in systemic sclerosis-associated pulmonary hypertension: A cross-sectional study. *J Scleroderma Relat Disord* 2023; 8: 221-230. 20230522. DOI: 10.1177/23971983231175213.

32. Pulito-Cueto V, Remuzgo-Martinez S, Genre F, et al. E-Selectin, ICAM-1, and ET-1 Biomarkers Address the Concern of the Challenging Diagnosis of Interstitial Lung Disease in Patients with Autoimmune Diseases. *Int J Mol Sci* 2023; 24 20230807. DOI: 10.3390/ijms241512518.
