# Supplementary figures and images for: Endothelin-1 as a Candidate Biomarker of Systemic Sclerosis: A GRADE-Assessed Systematic Review and Meta-Analysis With Meta-Regression
Source: Biomark Insights. 2025 Feb 21;20:11772719251318555. doi: 10.1177/11772719251318555 (PMC11846126; doi:10.1177/11772719251318555)

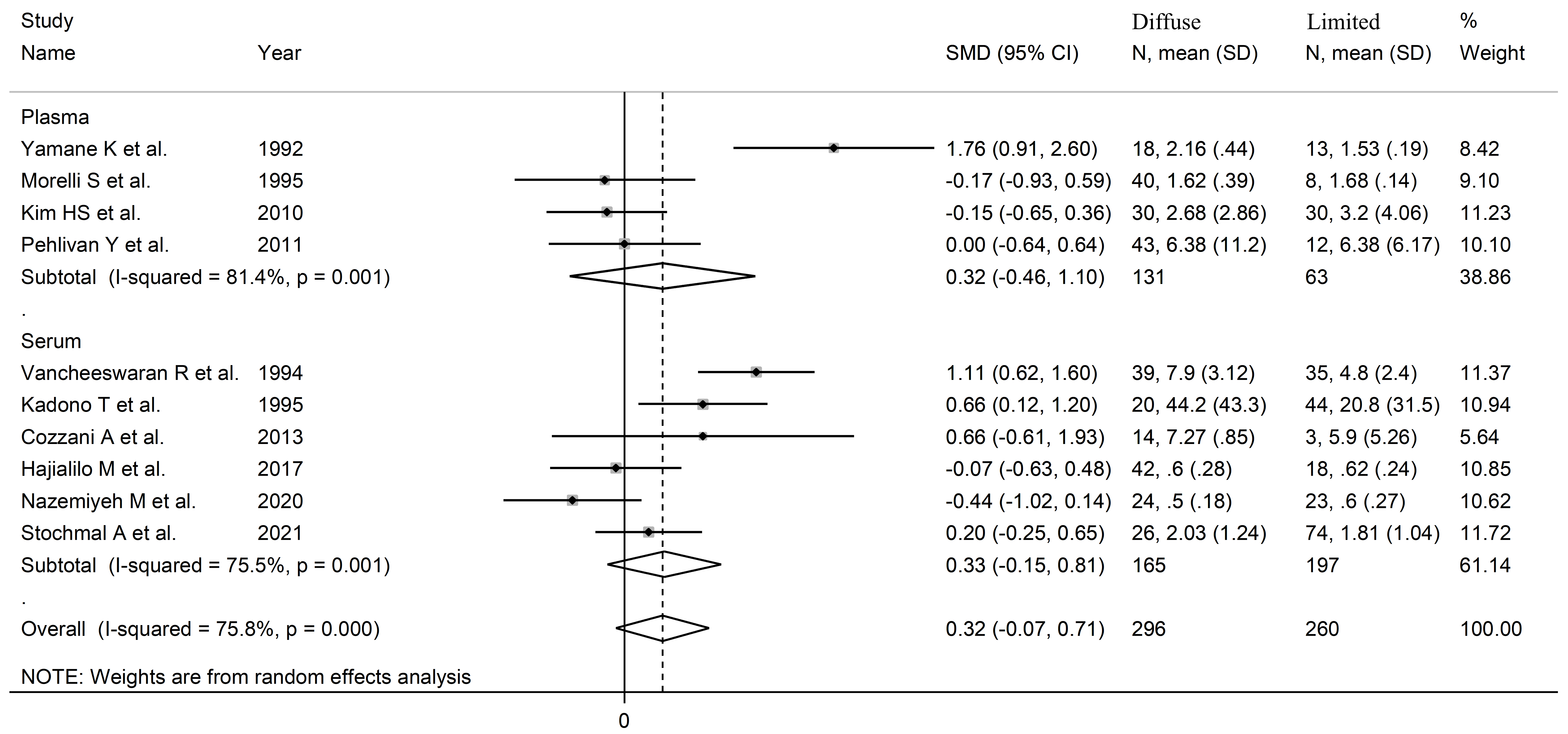

Supplement: sj-tif-10-bmi-10.1177_11772719251318555 – Supplemental material for Endothelin-1 as a Candidate Biomarker of Systemic Sclerosis: A GRADE-Assessed Systematic Review and Meta-Analysis With Meta-Regression [file sj-tif-10-bmi-10.1177_11772719251318555.tif]

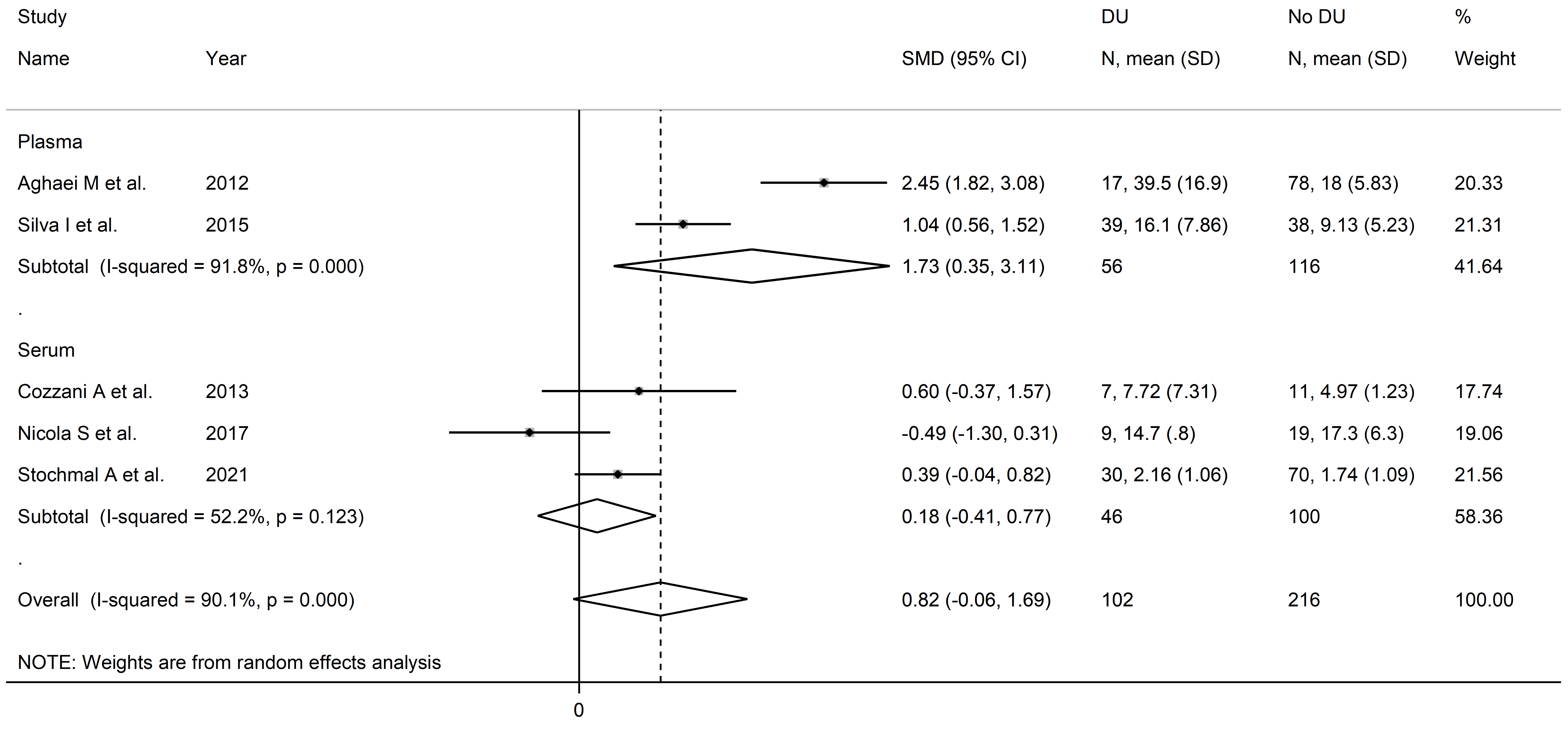

Supplement: sj-tif-11-bmi-10.1177_11772719251318555 – Supplemental material for Endothelin-1 as a Candidate Biomarker of Systemic Sclerosis: A GRADE-Assessed Systematic Review and Meta-Analysis With Meta-Regression [file sj-tif-11-bmi-10.1177_11772719251318555.tif]

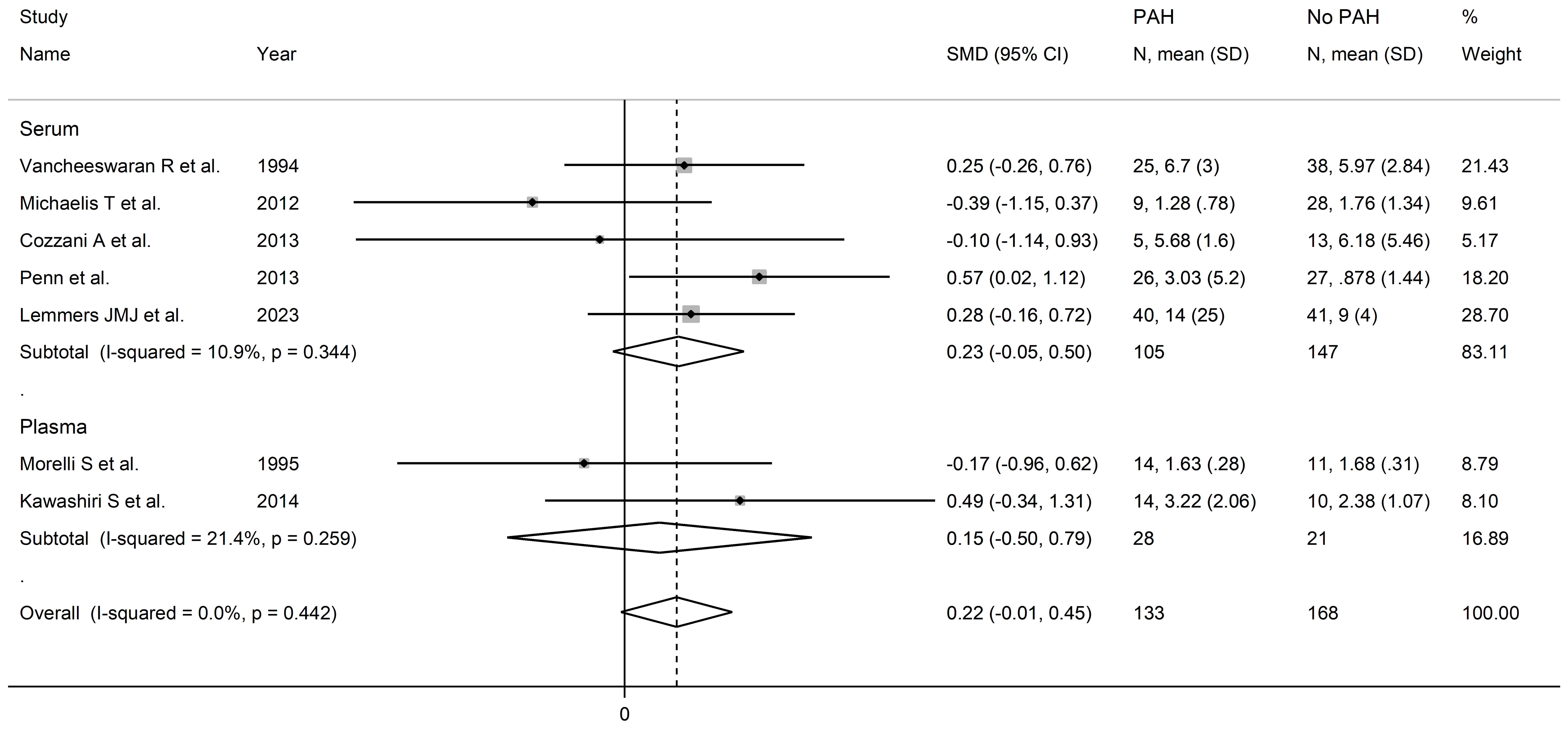

Supplement: sj-tif-12-bmi-10.1177_11772719251318555 – Supplemental material for Endothelin-1 as a Candidate Biomarker of Systemic Sclerosis: A GRADE-Assessed Systematic Review and Meta-Analysis With Meta-Regression [file sj-tif-12-bmi-10.1177_11772719251318555.tif]

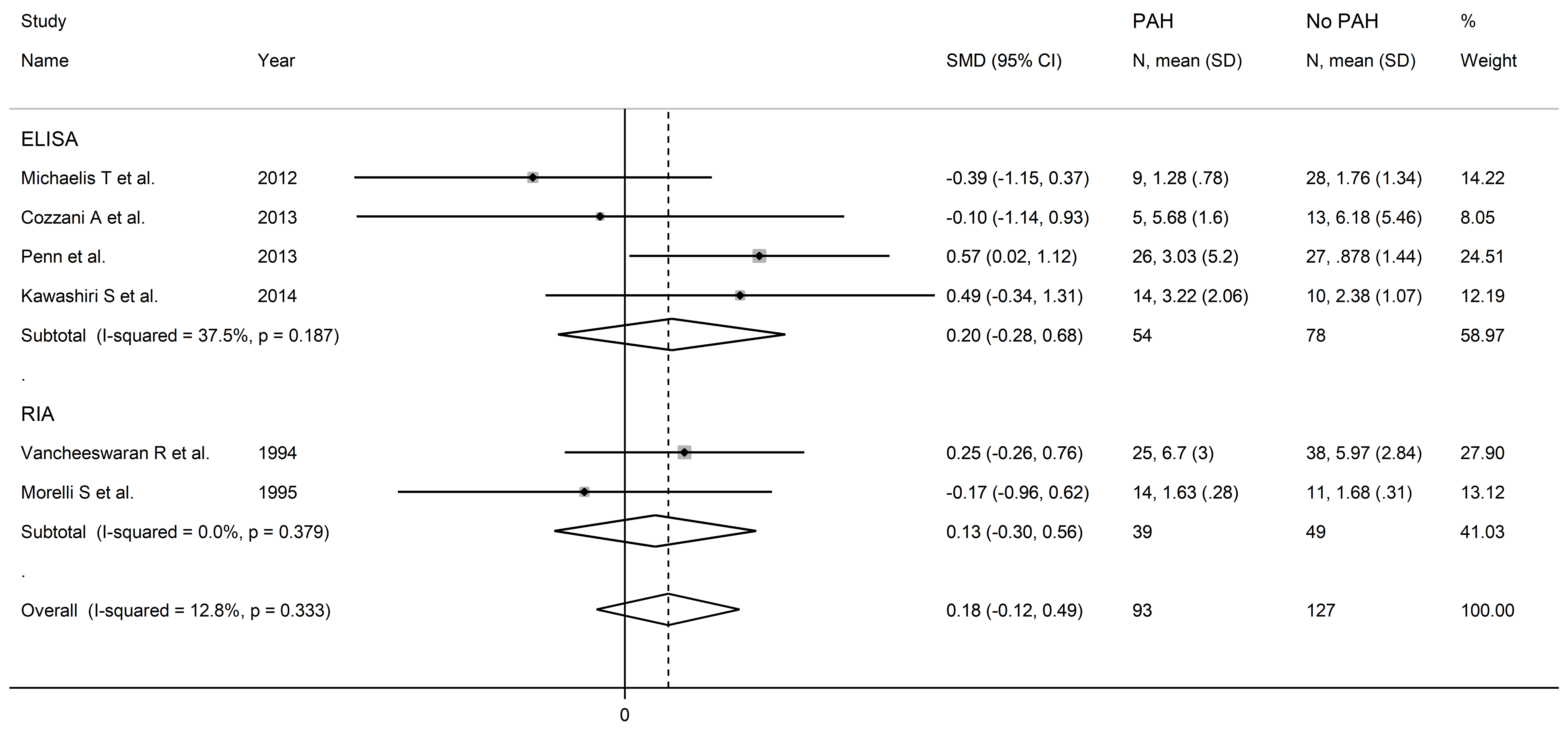

Supplement: sj-tif-13-bmi-10.1177_11772719251318555 – Supplemental material for Endothelin-1 as a Candidate Biomarker of Systemic Sclerosis: A GRADE-Assessed Systematic Review and Meta-Analysis With Meta-Regression [file sj-tif-13-bmi-10.1177_11772719251318555.tif]

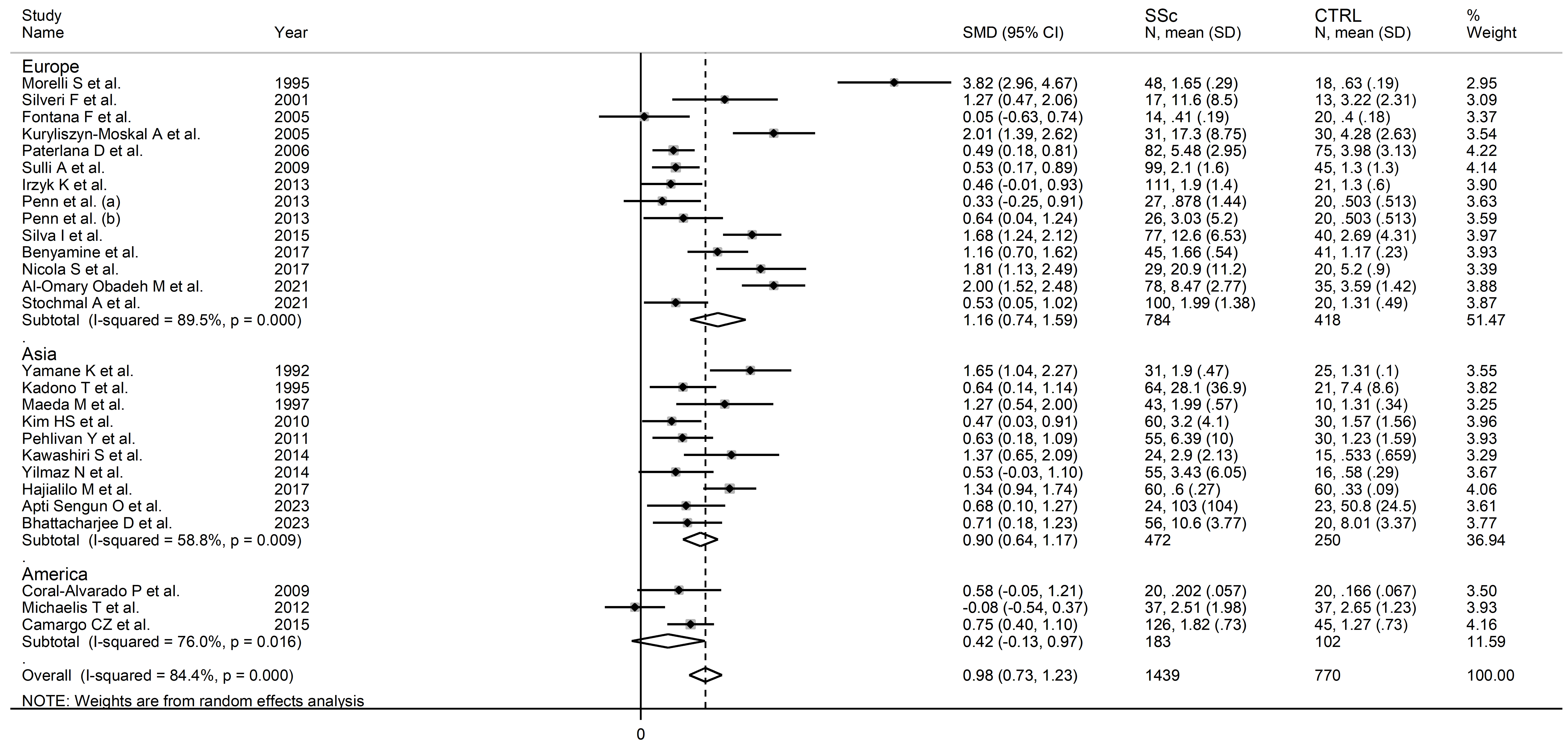

Supplement: sj-tif-4-bmi-10.1177_11772719251318555 – Supplemental material for Endothelin-1 as a Candidate Biomarker of Systemic Sclerosis: A GRADE-Assessed Systematic Review and Meta-Analysis With Meta-Regression [file sj-tif-4-bmi-10.1177_11772719251318555.tif]

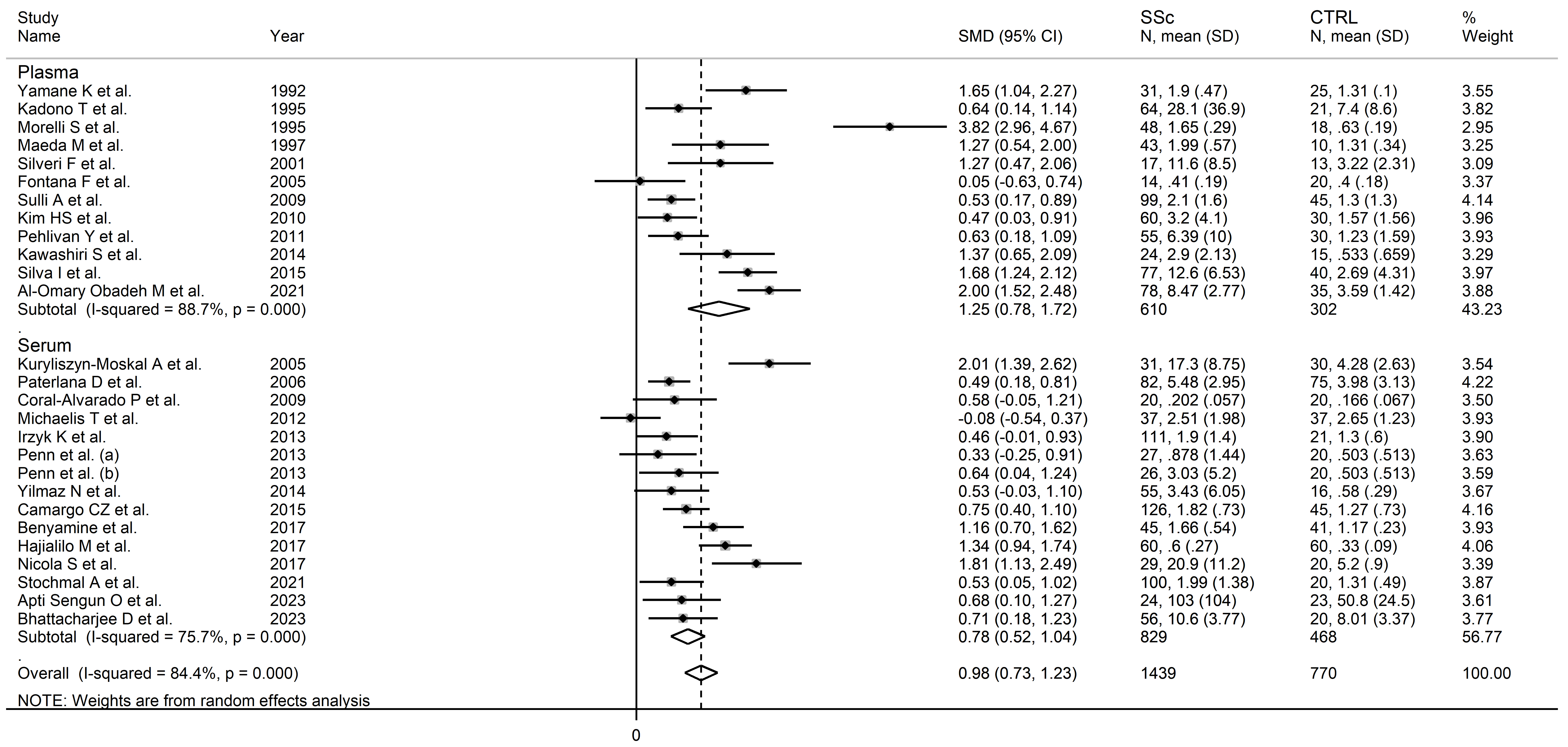

Supplement: sj-tif-5-bmi-10.1177_11772719251318555 – Supplemental material for Endothelin-1 as a Candidate Biomarker of Systemic Sclerosis: A GRADE-Assessed Systematic Review and Meta-Analysis With Meta-Regression [file sj-tif-5-bmi-10.1177_11772719251318555.tif]

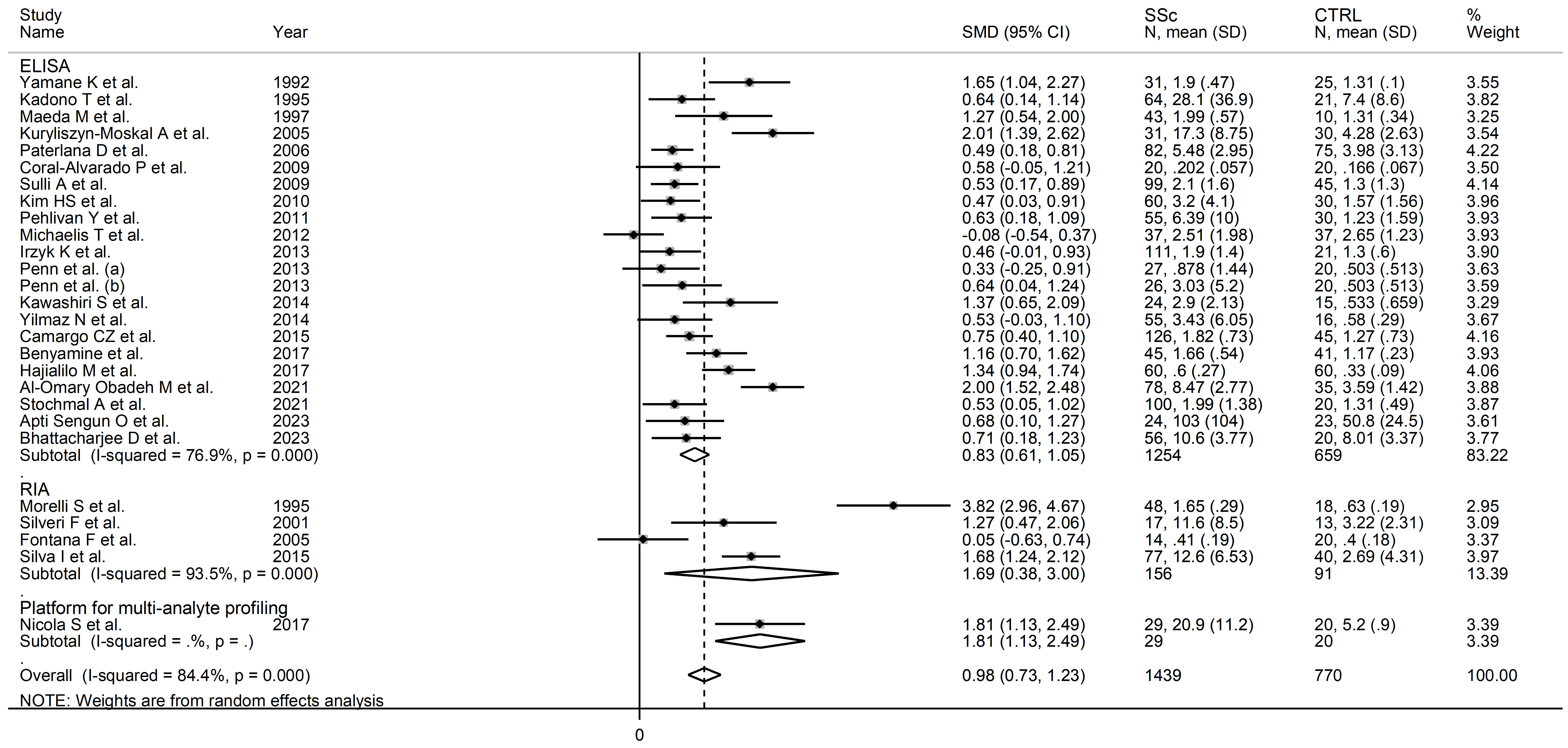

Supplement: sj-tif-6-bmi-10.1177_11772719251318555 – Supplemental material for Endothelin-1 as a Candidate Biomarker of Systemic Sclerosis: A GRADE-Assessed Systematic Review and Meta-Analysis With Meta-Regression [file sj-tif-6-bmi-10.1177_11772719251318555.tif]

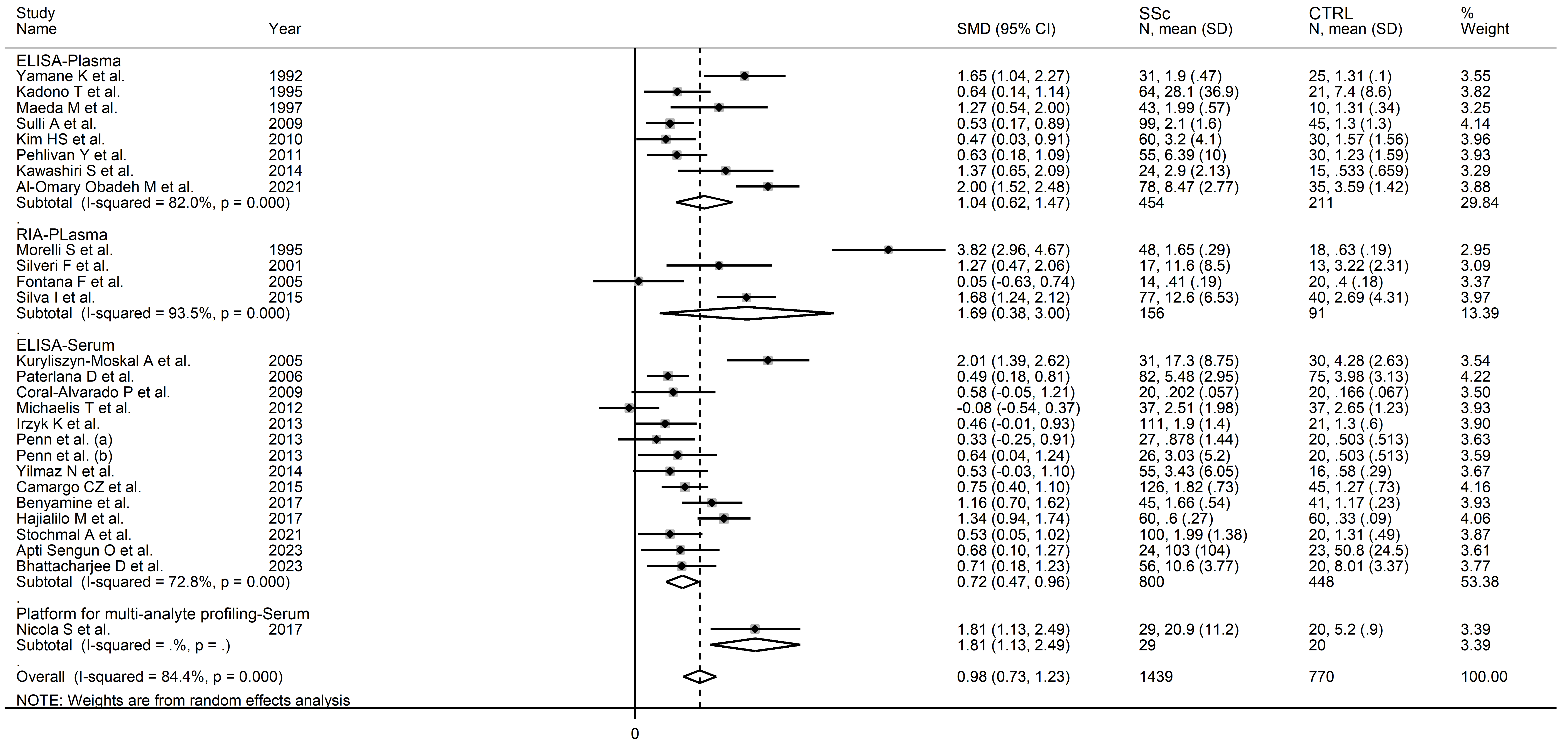

Supplement: sj-tif-7-bmi-10.1177_11772719251318555 – Supplemental material for Endothelin-1 as a Candidate Biomarker of Systemic Sclerosis: A GRADE-Assessed Systematic Review and Meta-Analysis With Meta-Regression [file sj-tif-7-bmi-10.1177_11772719251318555.tif]

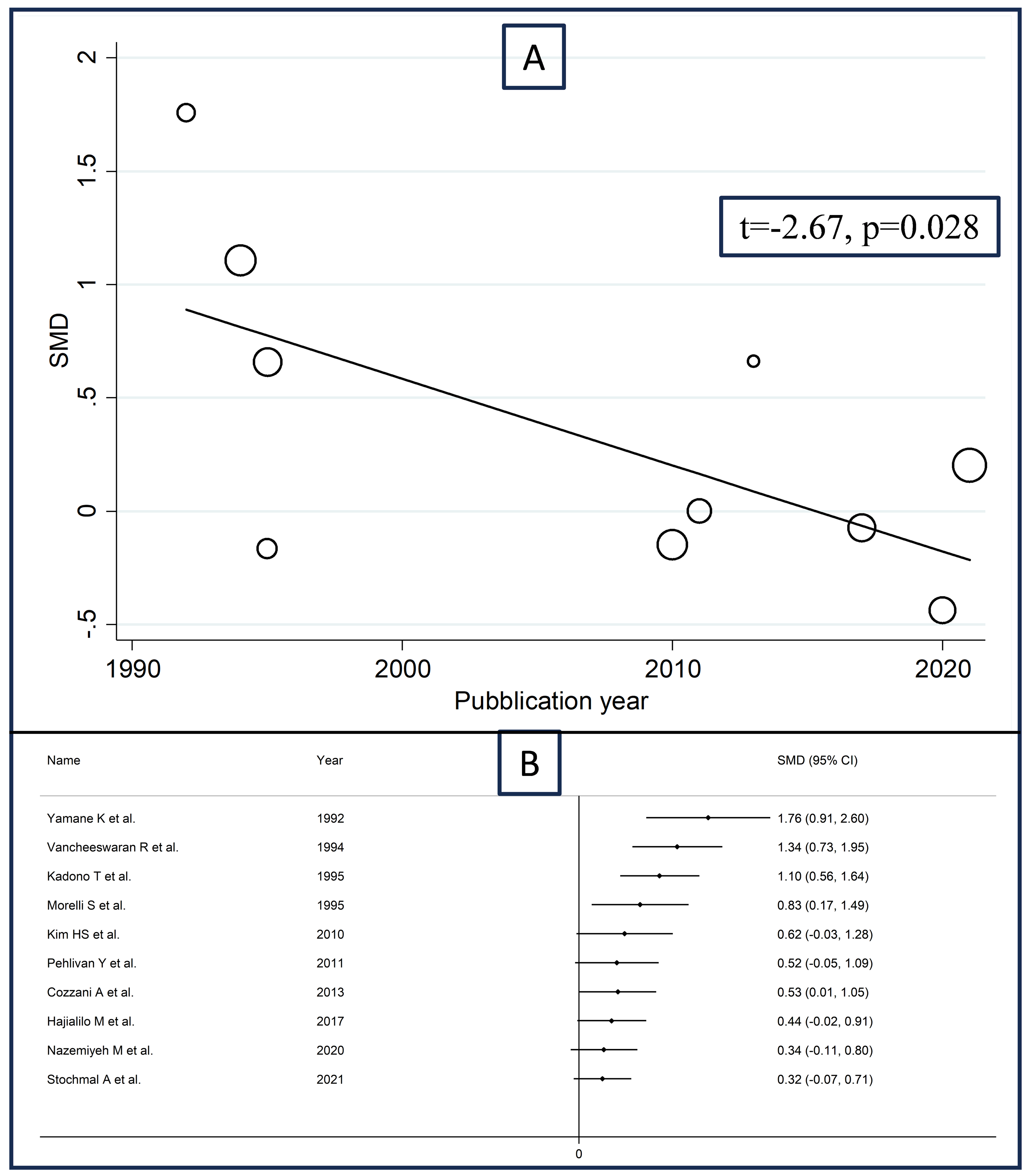

Supplement: sj-tif-8-bmi-10.1177_11772719251318555 – Supplemental material for Endothelin-1 as a Candidate Biomarker of Systemic Sclerosis: A GRADE-Assessed Systematic Review and Meta-Analysis With Meta-Regression [file sj-tif-8-bmi-10.1177_11772719251318555.tif]

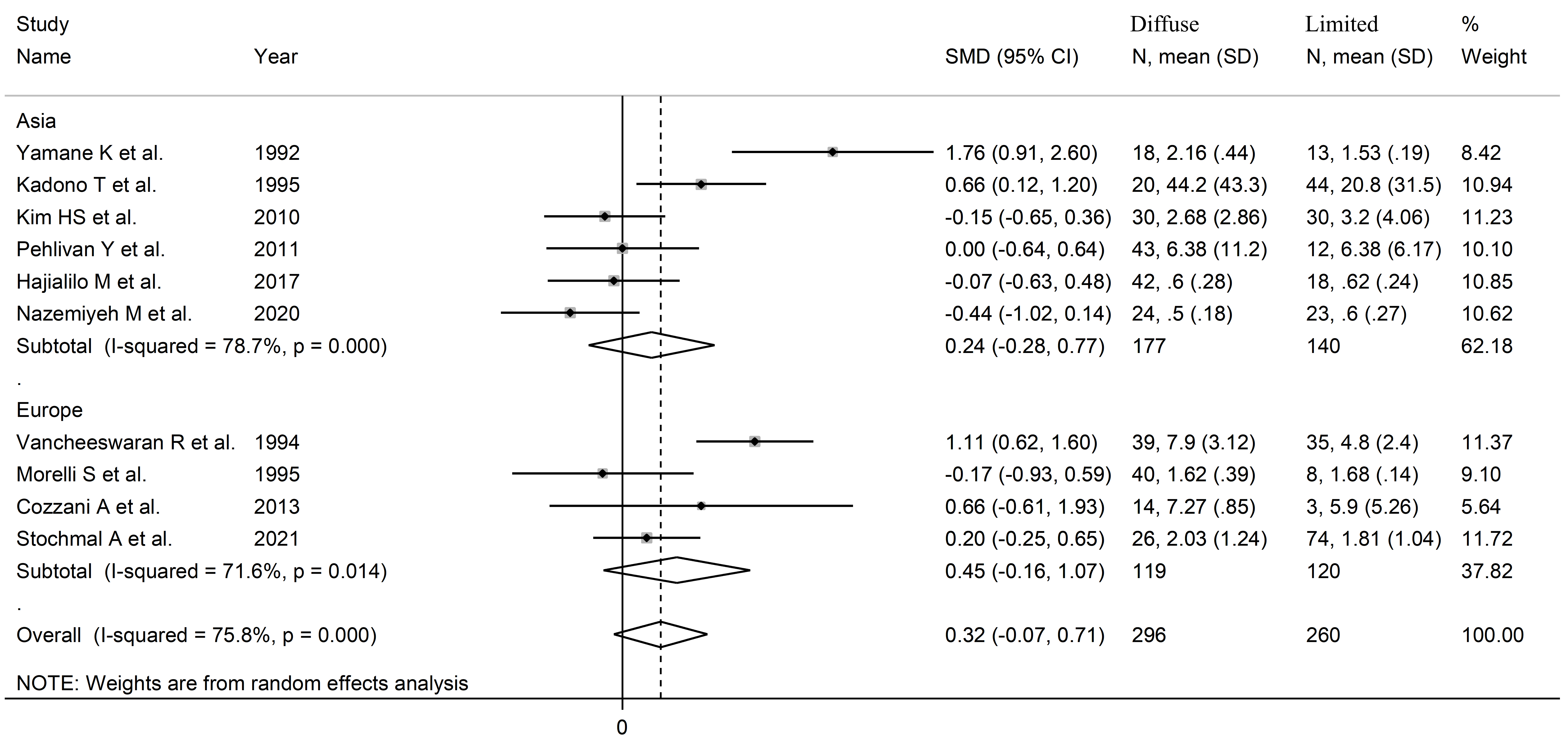

Supplement: sj-tif-9-bmi-10.1177_11772719251318555 – Supplemental material for Endothelin-1 as a Candidate Biomarker of Systemic Sclerosis: A GRADE-Assessed Systematic Review and Meta-Analysis With Meta-Regression [file sj-tif-9-bmi-10.1177_11772719251318555.tif]
